# Rabconnectin-3a Regulates Vesicle Endocytosis and Canonical Wnt Signaling in Zebrafish Neural Crest Migration

**DOI:** 10.1371/journal.pbio.1001852

**Published:** 2014-05-06

**Authors:** Adam M. Tuttle, Trevor L. Hoffman, Thomas F. Schilling

**Affiliations:** Department of Developmental and Cell Biology, University of California, Irvine, California, United States of America; The Wellcome Trust Sanger Institute, United Kingdom

## Abstract

Cell migration requires dynamic regulation of cell–cell signaling and cell adhesion. Both of these processes involve endocytosis, lysosomal degradation, and recycling of ligand–receptor complexes and cell adhesion molecules from the plasma membrane. Neural crest (NC) cells in vertebrates are highly migratory cells, which undergo an epithelial–mesenchymal transition (EMT) to leave the neural epithelium and migrate throughout the body to give rise to many different derivatives. Here we show that the v-ATPase interacting protein, Rabconnectin-3a (Rbc3a), controls intracellular trafficking events and Wnt signaling during NC migration. In zebrafish embryos deficient in Rbc3a, or its associated v-ATPase subunit Atp6v0a1, many NC cells fail to migrate and misregulate expression of cadherins. Surprisingly, endosomes in Rbc3a- and Atp6v0a1-deficient NC cells remain immature but still acidify. Rbc3a loss-of-function initially downregulates several canonical Wnt targets involved in EMT, but later Frizzled-7 accumulates at NC cell membranes, and nuclear B-catenin levels increase. Presumably due to this later Wnt signaling increase, Rbc3a-deficient NC cells that fail to migrate become pigment progenitors. We propose that Rbc3a and Atp6v0a1 promote endosomal maturation to coordinate Wnt signaling and intracellular trafficking of Wnt receptors and cadherins required for NC migration and cell fate determination. Our results suggest that different v-ATPases and associated proteins may play cell-type-specific functions in intracellular trafficking in many contexts.

## Abstract 2

A novel role for Rabconnectin-3a in endosome maturation regulates Wnt signaling, migration, and fate specification in zebrafish neural crest cells.

## Author Summary

The neural crest is a highly migratory population of embryonic cells, which requires Wnt signaling at several stages to promote migration and cell fate decisions. Intracellular trafficking of Wnt receptors and associated proteins can affect the timing and intensity of Wnt signaling. An obvious question is whether proton pumps and/or their partner proteins that are associated with intracellular vesicles might have a role in intracellular trafficking, Wnt signaling, and cell migration/adhesion. In this study we demonstrate such a role for Rabconnectin-3a, a protein associated with the vacuolar-ATPase (v-ATPase) proton pump complex. Loss of Rabconnectin-3a in zebrafish embryos disrupts the maturation of endocytic vesicles in neural crest cells, which has two effects: (1) decreasing Wnt signaling in these cells before migration and (2) increasing Wnt signaling after migration. Prior to migration, endosomes that fail to mature reduce Wnt signaling in neural crest cells and disrupt the localization and expression of cadherins, membrane-bound cell adhesion molecules required for these cells to initiate an epithelial-mesenchymal transition. At later stages, however, Wnt receptors accumulate at the membranes of unmigrated neural crest cells due to defective endocytosis, which correlates with high levels of Wnt signaling. Interestingly, Rabconnectin-3a-deficient neural crest cells that fail to migrate become pigment cells, presumably due to elevated Wnt signaling. Rabconnectin-3a may have a conserved role in endosomal maturation, Wnt signaling, and cell migration in many other cell populations.

## Introduction

The epithelial-mesenchymal transition (EMT) is characterized by loss of epithelial cell adhesion, dynamic expression and subcellular localization of cell–cell adhesion molecules, and increased cell motility [1]. EMT is a hallmark of cancer metastasis [2] and of many cell populations during embryogenesis. Neural crest (NC) cells in vertebrate embryos undergo a classic EMT to separate from the neural ectoderm [3] and become highly migratory progenitor cells, which give rise to a wide variety of cell types including cartilage and bone of the craniofacial skeleton, peripheral neurons/glia, and pigment cells [4]. NC cells initiate EMT in response to a variety of external signals, but how these are integrated spatially and temporally to give rise to different NC cell populations is poorly understood.

Proper timing of signal transduction [5] and dynamic expression and subcellular localization of adhesion molecules is required for NC EMT and migration [6]–[9]. Secreted Wnt ligands bind to Frizzled (Fz) receptors to promote canonical Wnt signaling by stabilizing B-catenin (Bcat) in the cytosol and allowing it to translocate to the nucleus and regulate target gene expression [10]. In NC cells this promotes EMT through downstream gene targets such as the transcription factors *Snail* [11], *Slug* [12], and *Twist* [13]. These transcription factors regulate many of the cellular changes required for EMT such as downregulation of epithelial cadherins (e.g., *Ecad*, *Ncad*) and subsequent upregulation of more “mesenchymal” cadherins (e.g., *Cdh6*, *Cdh7*) [6]. Disrupting Wnt signaling prior to EMT prevents NC delamination and migration in chick [14], and reducing expression of *Snail* or *Twist1* prevents early NC migration in *Xenopus* [15]. Canonical Wnt signaling also determines trunk NC cell fate at later stages by driving a pigment progenitor fate at the expense of neuronal/glial fates, both in zebrafish [16] and mouse [17].

Endocytosis and intracellular trafficking of Fz modulates Wnt signaling [18],[19]. Moreover, formation of endocytic vesicular compartments containing Wnt-bound Fz receptors, termed “signalosomes,” can impact the intensity and response of the Wnt signal within a cell [20]. Regulation of endosomal pH can also attenuate Wnt and other signaling pathways. Intracellular vesicles acidify by the recruitment and activity of the vacuolar-ATPase (v-ATPase) complex, which pumps protons into the lumen of vesicles, lowering their pH. Adaptor proteins such as Prorenin Receptor recruit the v-ATPase complex to endosomes containing Fz and its co-receptor LRP5/6 and regulate Wnt signaling through modulation of intracellular pH [21].

Rabconnectin-3a (Rbc3a) is a large, ∼325 kDa protein, highly conserved in multicellular organisms, which associates with its obligate binding partner, Rbc3b, and subunits of the v-ATPase complex [22],[23]. Studies in *Drosophila* embryos and murine cell culture have shown that Rbc3a and Rbc3b are required for proper lysosomal acidification and regulation of the Notch signaling pathway [24],[25]. In these cases, Rbc3a is required for v-ATPase function in endosomes/lysosomes and lowers their luminal pH. Low vesicular pH is necessary for γ-secretase activity in the lysosome, which cleaves the Notch receptor and allows translocation of the Notch intracellular domain to the nucleus to promote downstream Notch signaling. Mutations in *rbc3a* in zebrafish reduce synaptic vesicle acidification in hair cells, leading to aberrant responses to acoustic stimuli at 5 d postfertilization (dpf) [26]. Rbc3a promotes the polarized colocalization of the Atp6v0a1 (V0a1) and Atp6v1a1 (V1a1) v-ATPase subunits within these hair cells, allowing the acidification of synaptic vesicles required for optimal synaptic function.

However, v-ATPase subunits have other functions in cells besides vesicle acidification. For example, in microglial cells in the zebrafish central nervous system, disruption of the v-ATPase subunit isoform V0a1 causes a failure of autophagosomes to mature and fuse, but they acidify normally [27]. Additionally, when the fly ortholog of the V0a1 subunit, vha100-1, is deleted in fly photoreceptors, early endocytosis and recycling are disrupted, but endosomes still acidify [28]. Thus v-ATPase subunits may alter cell–cell signaling, such as Wnt signaling, through acidification-independent mechanisms, which may be shared with other partner proteins associated with intracellular vesicles such as Rbc3a.

Here we show that both zebrafish Rbc3a and V0a1 control intracellular trafficking events required for Wnt signaling during NC migration. *rbc3a* is highly expressed in premigratory NC cells. Embryos deficient in Rbc3a or V0a1 show NC-specific defects in endosomal maturation, expression of pro-EMT genes, and subsequent migration, which are acidification-independent. Wnt signaling is initially downregulated in these embryos but later rebounds, which correlates with the fact that unmigrated NC cells in Rbc3a-deficient embryos become pigment progenitors and not other NC cell types. We propose that Rbc3a promotes maturation and fusion of endosomes to regulate Wnt signaling in NC cells by altering the intracellular trafficking of receptors.

## Results

### *rbc3a* Is Expressed in Premigratory NC

**Figure 1:**
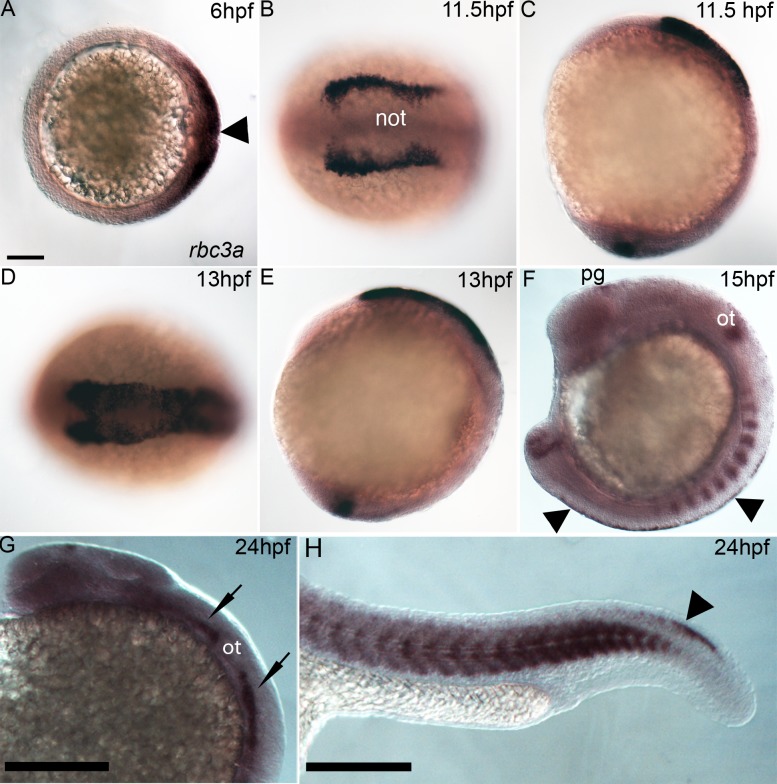
*rbc3a* expression. Whole mount in situ hybridization for *rbc3a* mRNA, anterior to the left. (A) Dorsal expression at gastrulation onset (6 hpf) includes the embryonic shield (arrowhead). Animal pole view, dorsal to the right. (B–E) Expression in NC cells starts at premigratory stages,11.5 hpf (B, C) and 13 hpf (D, E), with lower levels in the presumptive notochord (not) and in the tailbud (C, E). Dorsal views (B, D); lateral views (C, E). (F) Expression at 15 hpf in the pineal gland (pg), otic vesicle (ot), somites, tailbud, and premigratory NC (arrowheads) in the trunk. (G, H) Expression at 24 hpf in the pineal gland, cranial sensory ganglia (black arrows), somites, and tail NC (arrowhead). Scale bars, 100 µm.

Zebrafish *rbc3a*, also annotated *dmxl2*, was identified in a microarray screen with *tfap2a/g*-deficient embryos at 12 hpf, which completely lack NC cells [29]. The full-length open-reading frame (ORF) of *rbc3a* was cloned from 12 hpf *D. rerio* cDNA (Genbank No. KF147926). Zebrafish *rbc3a* expression was described in larval hair cells, but earlier embryonic expression and function was not examined [26]. Whole mount *in situ* hybridization first detected *rbc3a* expression on the dorsal side of the gastrula (6 hpf), including the shield organizer (Figure 1A). By 11.5 hpf, expression was strong in the premigratory NC, first in the cranial region (Figure 1B–E), and by 15 hpf along the entire trunk and tail NC (Figure 1F,H). Expression was also detected in the tailbud (Figure 1C,E,F), somites (Figure 1F,H), cranial sensory ganglia (Figure 1G), and pineal gland (Figure 1F,G).

### Rbc3a Knockdown Disrupts Migration of a Subset of NC

**Figure 2:**
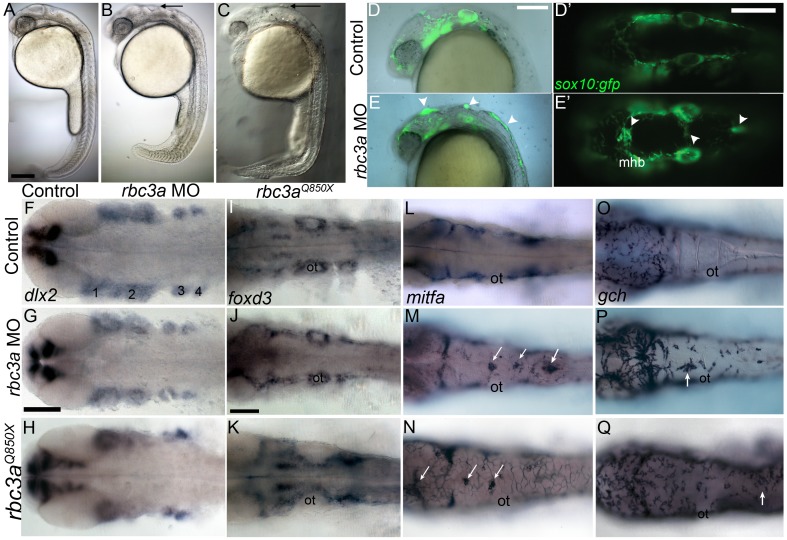
Rbc3a loss of function disrupts NC migration. (A–C) Live 24 hpf controls (A), *rbc3a*-MO1–injected (B), and *rbc3a^Q850X^* mutant (C) embryos. Morphant/mutant morphological defects include the mhb and cell aggregates in the dorsal midline (black arrows in B and C). (D–E′) NC defects in live *sox10:gfp* transgenics injected with *rbc3a*-MO1. (D, E) Merged bright-field and fluorescent images of the cranial region at 24 hpf, lateral views, show GFP+ NC cells accumulated dorsally (white arrowheads). (D′, E′) Dorsal views showing midline position of aggregates over the mhb and further posteriorly (white arrowheads). (F–Q) Whole mount in situ hybridization for markers of different NC lineages in controls (row 1), *rbc3a*-MO1–injected (row 2), and *rbc3a^Q850X^* mutants (row 3) at 28 hpf, dorsal views, anterior to the left. *dlx2* (F–H) expression in skeletogenic NC and *foxd3* (I–K) expression in gliogenic NC appear unaffected, while *mitfa* in presumptive melanocytes (L–N) and *gch* in xanthophores (O–Q) are expressed in dorsal midline aggregates in *rbc3a* morphants/mutants (white arrows). Abbreviations: 1–4, pharyngeal arches; ot, otic vesicle; mhb, midbrain-hindbrain boundary. Scale bars, 100 µm.

Knockdown of *rbc3a* via microinjection of a translation-blocking *rbc3a* antisense morpholino oligonucleotide (*rbc3a*-MO1; 2.0 ng/embryo) targeted to the 5′ UTR produced a shortened, kinked tail and disrupted morphology of the midbrain-hindbrain boundary (MHB) at 24 hpf (Figure 2B,E). Zebrafish embryos homozygous for the *rbc3a* mutant allele *stardust (rbc3a* ^Q850X^) showed similar tail curvature and MHB defects (Figure 2C). By 72 hpf, both mutant and *rbc3a* MO1-injected embryos exhibited defects in melanocyte pigmentation, particularly in the trunk and tail, a kink at the head/trunk boundary, and shortened, curved tails with reduced ventral tail fins (Figure S1).

*rbc3a*-MO1 injections into transgenic *Tg(7.2 kb-sox10:gfp)*—hereafter referred to as *sox10:gfp*—embryos (which labels NC cells) revealed GFP+ dorsal midline cell aggregates at 24 hpf, which appeared to be NC cells that failed to migrate (Figure 2D–E′). Co-injection of 100 pg/embryo of full-length, *in vitro* transcribed *rbc3a* mRNA with *rbc3a*-MO1 significantly reduced the number of NC cells remaining in the dorsal midline (Figure S2). Injection of a second, translation-blocking MO targeting the first ATG in the ORF of *rbc3a* (*rbc3a*-MO2, 5 ng/embryo) produced similar defects in the distribution of *sox10:GFP*+ cells (Figure S3; Table S1; *n* = 35/41 embryos; X^2^ = 47.5, *p*≪0.001), which were also partially rescued by co-injection with full-length *rbc3a* mRNA, confirming specificity (Table S1; *n* = 11/21, X^2^ = 9.94, *p* = 0.0016). Similar controls with *rbc3a*-MO2 were not performed for subsequent experiments. Hereafter, unless otherwise noted, all Rbc3a knockdown experiments were performed with the *rbc3a*-MO1. Additionally, injection of a truncated *Xenopus tropicalis rbc3a* DNA construct (Open Biosystems, Accession No. BC127555) missing the 3′ ∼2,200 bp, which contains several predicted WD40-repeat domains, into *sox10:gfp* embryos produced similar NC migration defects (Figure S4). Co-injection of this construct with subthreshold levels (1.0 ng/embryo) of *rbc3a*-MO1 had a synergistic effect, causing larger numbers of dorsal NC aggregates per embryo, suggesting that it acts as a dominant negative.

To determine if cells that aggregate in the dorsal midline of embryos injected with *rbc3a-*MO1 were NC cells that failed to migrate, we performed time-lapsed confocal imaging of control and *rbc3a*-MO1–injected *sox10:gfp* embryos. Early premigratory NC appeared to form normally at 12 hpf and began to extend filopodia in *rbc3a*-MO1–injected embryos, but by 16 hpf, many of these cells remained in distinct patches of GFP+ cells at the dorsal midline that were never seen in wild-type, *sox10:gfp* controls (Movies S1 and S2 and Figure S5). In contrast to wild-type NC cells (3 embryos, *n* = 12), NC cells in *rbc3a*-MO1–injected embryos (3 embryos, *n* = 11) showed significant reductions in migration speed (Figure S5M, *p*<0.001) and directionality/persistence (Figure S5N, *p*<0.01—distance between initial and final cell position divided by the total distance traveled). These results suggest that NC cells in *rbc3a*-MO1–injected embryos are motile, albeit at significantly reduced velocity, and fail to migrate directionally.

To determine if Rbc3a is required cell autonomously in NC cells, we transplanted *rbc3a*-MO1–injected, *sox10:lyn-tdtomato* donor cells into wild-type hosts. A significant number of chimeras displayed midline NC aggregates (*n* = 9/17 embryos) compared to wild-type donor cells (*n* = 0/30; Chi-squared test—X^2^ = 12.48, *p*<0.001), indicating a cell-autonomous requirement for Rbc3a in NC (Figure S9D).

To determine the identities of NC cells that remain at the midline in *rbc3a*-MO1–injected embryos, we performed whole mount *in situ* hybridization with probes for different NC lineages in both *rbc3a*-MO1–injected embryos and *rbc3a* mutants. At 28 hpf, dorsal midline NC aggregates in these embryos expressed melanophore (*mitfa*) and xanthophore (*gch*) progenitor markers (Figure 2L–Q), but did not express markers for skeletogenic (Figure 2F–H, *dlx2*) or gliogenic (Figure 2I–K, *foxd3*) NC cells. These data suggest that the NC cells that fail to migrate in *rbc3a*-MO1–injected embryos and *rbc3a* mutant embryos become specified as pigment cells.

### Rbc3a Knockdown Disrupts Endocytosis But Not Endosome Acidification in NC

**Figure 3:**
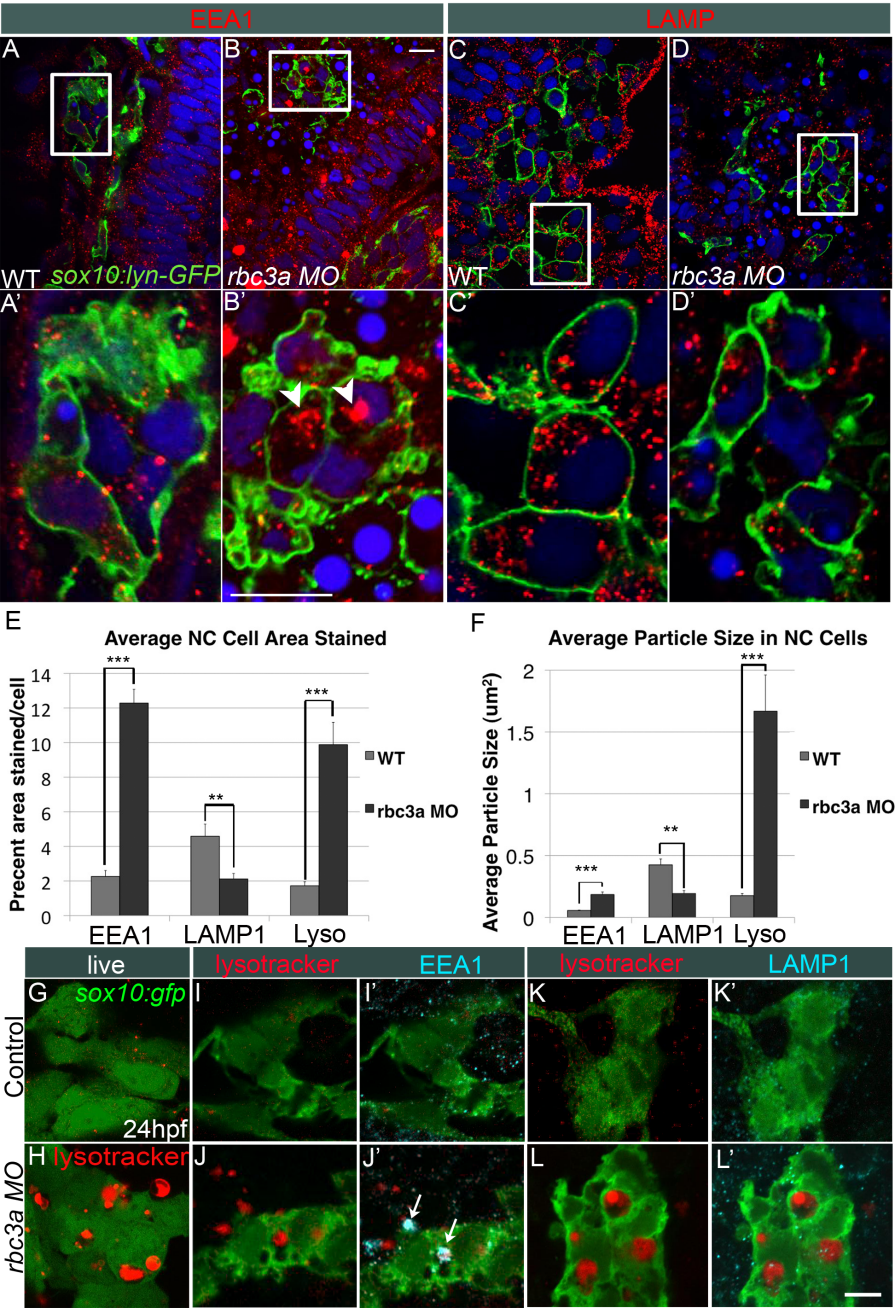
Rbc3a knockdown disrupts endosomal maturation but not acidification in NC. (A–D) Confocal images of whole mount immunohistochemical staining for endocytic markers in NC cells in *sox10:lyn-gfp* transgenics, which labels NC cell membranes (green). (A′–D′) 4× insets of (A–D). (A–B′) Anti-EEA1 marks early endosomes (red). *rbc3a*-MO1–injected embryos show large EEA1+ aggregates in NC cells (white arrowheads in B′). (C–D′) Anti-LAMP1 marks late endosomes/lysosomes (red). No increase in number or size of LAMP1+ vesicles was observed with *rbc3a*-MO1 injection. (E, F) Automated quantification of average % area (E) and average particle size (F) per NC cell stained positive for EEA1, LAMP1, or Lysotracker Red (Lyso) using ImageJ Particle Analyzer. *rbc3a-*MO1 injection produced significantly higher EEA1+ and Lysotracker+ relative area and particle size but significantly smaller LAMP1+ relative area and particle size per NC cell. Error bars represent ± SEM. (G, H) Live whole-mount images of *sox10:gfp*+ NC cells labeled with Lysotracker (red). *rbc3a*-MO1–injected embryos show many large, acidic Lyso+ vesicles, which colocalize with EEA1 (I′,J′, white arrows) but not LAMP1 (K′, L′). ** *p*<0.001, *** *p*<0.0001. Scale bar, 10 µm.

Rbc3a is known to associate with the v-ATPase complex and regulate lysosomal acidification in cells. Therefore, we examined the effects of Rbc3a knockdown on intracellular vesicle formation, acidification, and trafficking. In this case, we used *Tg(7.2 kb-sox10:lyn-gfp)—*hereafter referred to as *sox10:lyn-gfp*—trangenics, in which a membrane-localized variant of GFP was expressed in NC cells to avoid obscuring their intracellular organelles. Injection of *rbc3a*-MO1 led to the formation of large, clustered early endosomes within NC cells (*n* = 23 embryos), as determined by immunohistochemical staining with an antibody that recognizes Early Endosome Antigen 1 (EEA1) (Figure 3A–B′). However, surprisingly, these cells showed decreases in late endosomal/lysosomal size and number (*n* = 21 embryos), based on staining for Lysosomal Associated Membrane Protein 1 (LAMP1) (Figure 3C–D′). Automated analysis of intracellular EEA1 and LAMP1 staining in NC cells with ImageJ confirmed a significant increase in the percent area per NC cell stained positive for EEA1 in *rbc3a*-MO1–injected embryos (Figure 3E, *p*<0.001, *n* = 53 cells) with a corresponding decrease in LAMP1+ area (Figure 3E, *p*<0.001, *n* = 49 cells). Additionally, the average size of EEA1−, but not LAMP1−, stained intracellular particles was significantly larger in NC cells in *rbc3a*-MO1–injected embryos (Figure 3F, *p*<0.001). EEA1+ vesicles in midline NC cell aggregates increased in number and size in *rbc3a*-MO1–injected embryos over time, from 14 to 20 hpf, whereas these cells showed no difference in vesicle morphology using markers for recycling endosomes (antibody against Rab11a, Figure S6) or exocytic vesicles (antibody against Rab3ab, not shown). These results are in contrast to studies of Rbc3a orthologues in *Drosophila* embryos and murine cell culture [24],[25], where it is required for lysosomal morphology and acidification, and suggest instead that zebrafish Rbc3a plays a role in early endosome maturation.

Rbc3a is required for lysosomal acidification in many cells through its interactions with v-ATPase subunits. To measure vesicle acidity in NC cells following *rbc3a*-MO1 injection, the fixable vital dye Lysotracker Red DND-99 (Invitrogen), which labels acidic (pH<5.5) vesicles and lysosomes, was applied to *sox10:gfp* transgenics at 24 hpf. *rbc3a-*MO1–injected embryos showed an increase in number of large, Lysotracker-Red+ (Lyso+) intracellular vesicles in NC cells, most dramatically in NC cell aggregates at the dorsal midline (Figure 3H, *n* = 18 embryos) but also in some migrated NC cells within the pharyngeal arches (Figure S7B″). Automated analysis of intracellular Lysotracker staining in NC cells with ImageJ revealed a significant increase in percent area of cells (Figure 3E, *p*<0.001, *n* = 49 cells) and average particle size stained positive for Lysotracker (Figure 3F, *p*<0.001) in *rbc3a*-MO1–injected versus wild-type *sox10:gfp* embryos. Most Lyso+ vesicles in *rbc3a*-MO1–injected embryos co-localized with EEA1 (Figure 3I–J′, *n* = 12 embryos) but not LAMP1 staining (Figure 3K–L′, *n* = 11 embryos), suggesting the presence of abnormally large early endosomes or clusters of early endosomes, which were acidified. These results suggest that Rbc3a is required for endosome formation and maturation but not for their acidification.

### v-ATPase Subunit Isoform V0a1 Knockdown Phenocopies Rbc3a Depletion

Rbc3a promotes association of v-ATPase subunits. Loss of the v-ATPase subunit isoform ATP6V0A1 (v0a1) in zebrafish microglia [27] or the *Drosophila* ortholog vha100-1 in photoreceptors [28] produces a subcellular defect similar to our results with *rbc3a*-MO1 injection: that is, aggregation of early endosome/phagosomes that do not mature but still become acidified. Additionally, co-IP experiments with mouse Rbc3a show specific interactions with V0a1 but not other V0a subunit isoforms [23]. Therefore we tested requirements for V0a1 in vesicle trafficking and acidification during early NC migration.

**Figure 4:**
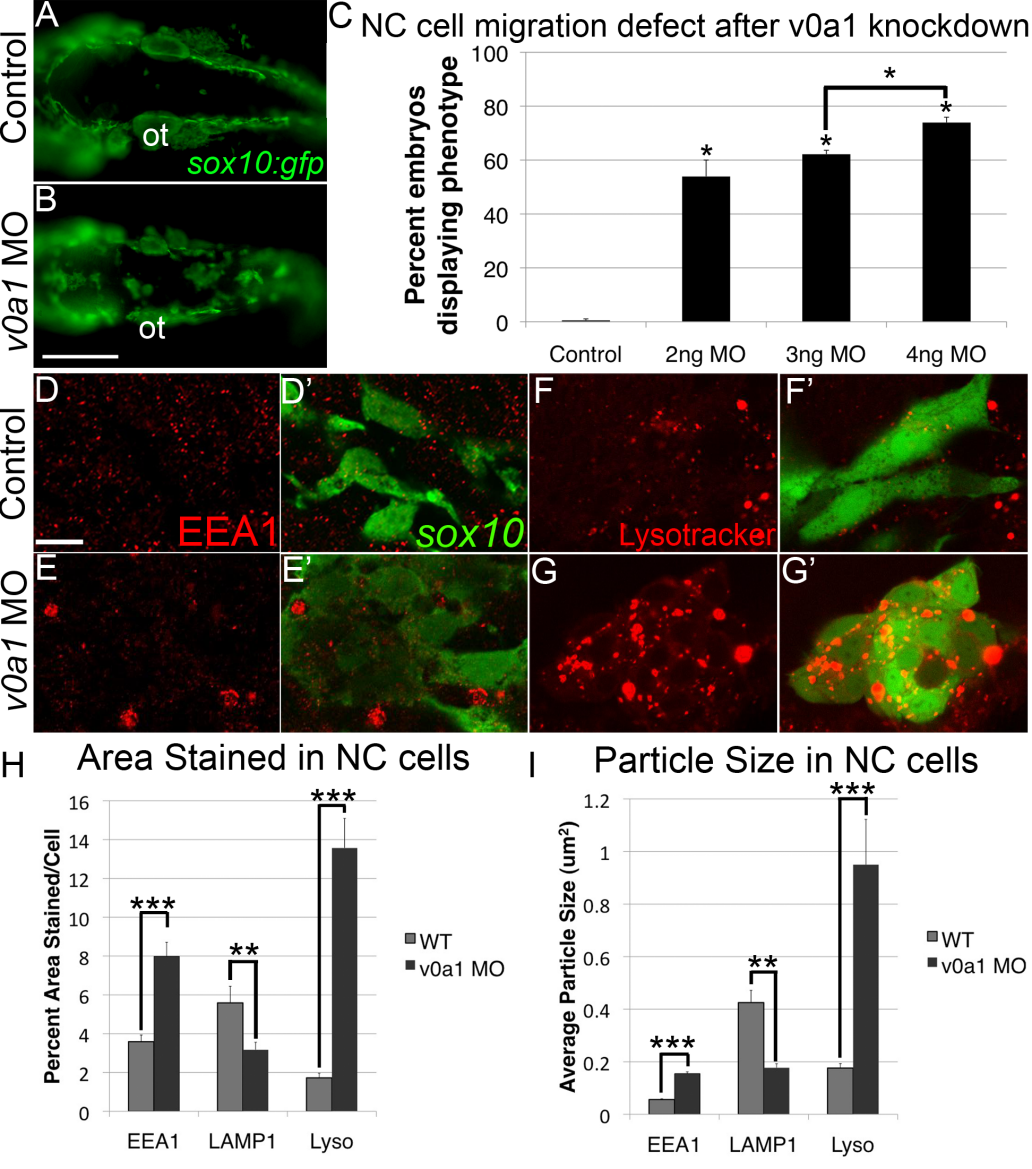
V0a1 knockdown disrupts NC migration and early endosome maturation. (A, B) Fluorescent images of live *sox10:gfp* embryos at 24 hpf, dorsal views, showing aggregates of GFP+ cells in the dorsal midline. Ot, otic vesicle. Scale bar, 100 µm. (C) Percentages of embryos with GFP+ aggregates in embryos injected with increasing amounts of *v0a1*-MO. (D–E′) EEA1 staining (red) in *sox10:gfp*+ NC cells (green), in controls (D, D′), and *v0a1*-deficient embryos (E, E′) showing EEA1+ vesicle enlargement at 24 hpf. Scale bar, 10 µm. (F–G′) Lysotracker staining (red) in *sox10:gfp+* NC cells in controls (F, F′) and V0a1-deficient embryos (G, G′) showing enlarged acidic intracellular compartments in NC cells. (H, I) Automated quantification of average % area (H) and average particle size (I) per NC cell stained positive for EEA1, LAMP1, or Lysotracker Red (Lyso) using ImageJ Particle Analyzer. V0a1 knockdown produced significantly higher EEA1+ and Lysotracker+ relative area and average particle size but significantly less LAMP1+ relative area and particle size per NC cell. Error bars represent ± SEM. * *p*<0.05, ** *p*<0.001, *** *p*<0.0001.

Injection of *sox10:gfp* embryos with a previously described translation-blocking *v0a1*-MO [27] caused a dose-dependent increase in dorsal midline aggregates of GFP+ cells, similar to *rbc3a*-MO1–injected embryos (Figure 4B,C, *n*>45 for each treatment). These *v0a1*-deficient NC cells also exhibited increased size and numbers of EEA1+ vesicles at 24 hpf (Figure 4E,E′). Automated analysis of intracellular EEA1 and LAMP1 staining in NC cells with ImageJ confirmed a significant increase in the percent area per cell stained positive for EEA1 in V0a1-deficient embryos (Figure 4H, *p*<0.001, *n* = 53 cells) but a significant decrease in percent area of cells stained positive for LAMP1 (Figure 4H, *p*<0.001, *n* = 61 cells). Additionally, the average size of EEA1+ particles was significantly larger and LAMP1-stained particles were significantly smaller in V0a1-deficient embryos (Figure 4I, *p*<0.001 in both cases).

Interestingly, knockdown of V0a1 produced enlarged Lyso+ vesicles in NC cells similar to those in *rbc3a*-MO1–injected embryos (Figure 4G,G′). Quantification of Lyso+ staining in NC cells confirmed a significant increase in relative area stained per NC cell in *v0a1*-deficient embryos compared with controls (Figure 4H, *p*<0.001, *n* = 69 cells). The average particle size of Lyso+ particles was also significantly larger in NC cells in *v0a1*-MO–injected embryos (Figure 4I, *p*<0.001). Taken together, these data suggest Rbc3a and V0a1 act together to regulate endocytosis and NC cell migration.

### Rbc3a or V0a1 Knockdown Disrupts Wnt Signaling During Early NC Migration

**Figure 5:**
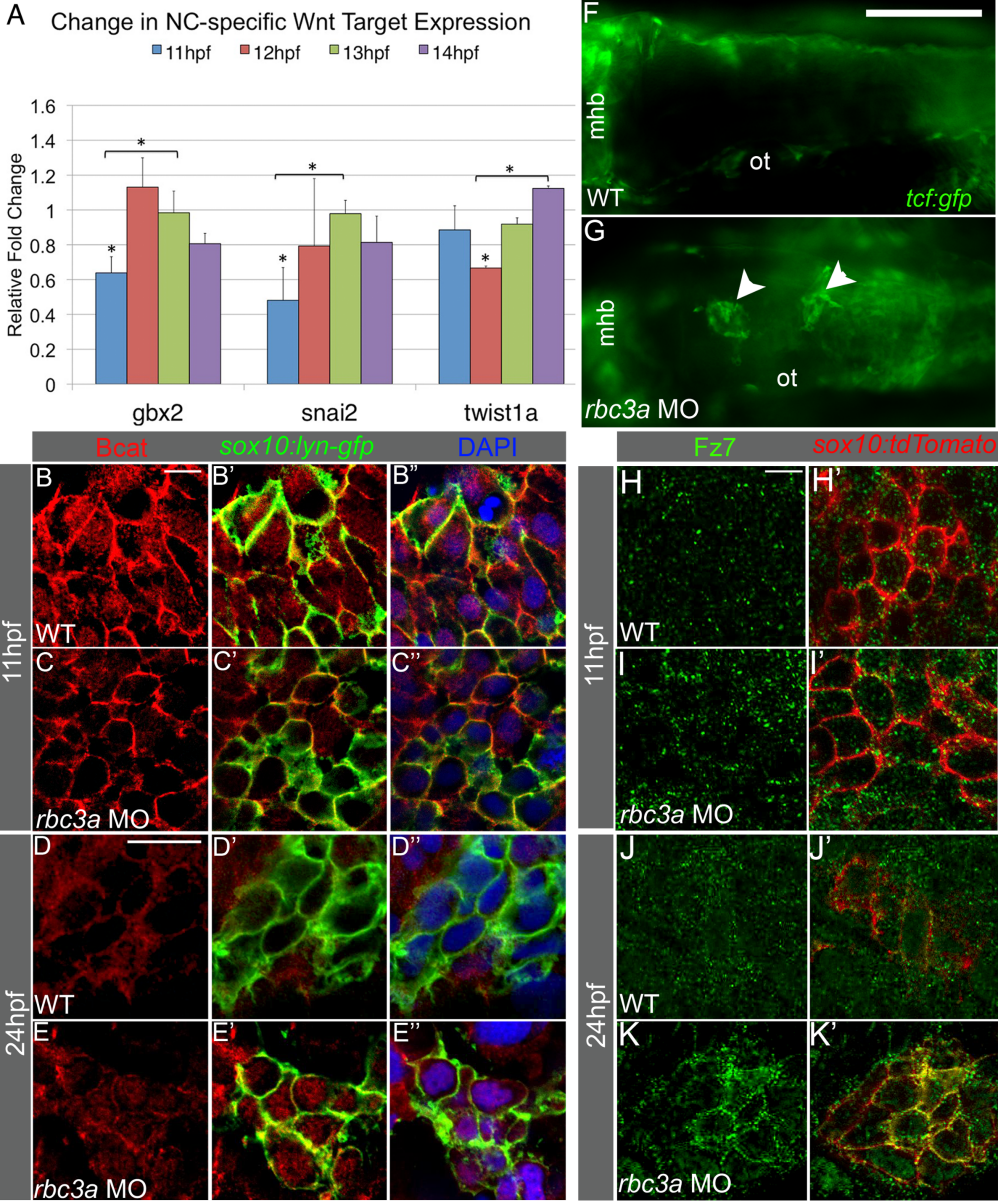
Rbc3a knockdown disrupts subcellular localization of Bcat and Fz7 in NC cells. (A) Quantitative RT-PCR analysis of early Wnt target genes with important roles in EMT reveals reduced expression of *snai2*, *gbx2*, and *twist1a* at 11–12 hpf in *rbc3a*-MO1–injected embryos. Error bars represent triplicate experiments ± SEM. * *p*<0.05. (B–E″) Immunostaining with an anti-Bcat antibody (red) in whole-mounted, *sox10:lyn-gfp* transgenic embryos to label NC cell membranes (green) and DAPI to label nuclei (blue). Bcat levels in the nucleus are (B–C″) reduced in *rbc3a*-MO1–injected embryos at 11 hpf and (D–E″) elevated in the nucleus of MO-injected embryos at 24 hpf compared with wild-type (WT) controls. Scale bar, 10 µm. (F, G) Dorsal images of *tcf:gfp* Wnt reporter transgenic fish at 24 hpf identifies distinct aggregates of GFP+ cells (white arrowheads) in the dorsal midline of *rbc3a*-MO1–injected embryos (G) but not wild-type (F) embryos. Mhb, midbrain-hindbrain boundary; ot, otic vesicle. Scale bar, 100 µm. (H–K′) Immunohistochemical staining for GFP (green) after microinjection of *fz7-yfp* mRNA in *sox10:lyn-tdtomato* (red) transgenic embryos. At both 11 hpf (H–I′) and 24 hpf (J–K′), the number of YFP+ puncta per cell increased in NC cells in *rbc3a*-MO1–injected embryos. YFP colocalizes with tdTomato at the membranes of NC cells in *rbc3a*-MO1–injected embryos at 24 hpf (K′). Scale bar, 10 µm.

Endocytosis and intracellular vesicle acidification have important roles in modulating cellular signaling pathways, including canonical Wnt signaling [18]. Because Wnt signaling influences NC induction, migration, and lineage specification [14],[16],[30], we examined changes in expression of several direct downstream targets of canonical Wnt signaling, which have roles in NC migration (Figure 5A). Quantitative PCR from whole embryos following injection of *rbc3a*-MO1 revealed significant reductions in expression of *gastrulation brain homeobox 2* (*gbx2*) and *snail2* (*snai2*) compared to wild-type, at 11 hpf (*p* = 0.017 and *p* = 0.008, respectively), which returned to normal levels by 13 hpf (*p* = 0.0097 and *p* = 0.0043, respectively). *twist1a*, a downstream target of *gbx2* and *snai2* transcription factors, was also significantly downregulated at 12 hpf (*p*<0.001) and this recovered by 14 hpf (*p* = 0.011). Decreases in *gbx2*, *snai2*, *twist1a*, and *axin2* were confirmed with *in situ* hybridization (Figure S8A–H). Additional direct canonical Wnt targets, including *axin2* and *nmyc*, were significantly downregulated at 13 hpf (Figure S8I, *p* = 0.006, *p* = 0.011, respectively) and *nmyc* and *axin2* expression returned to control levels by 14 hpf (*p* = 0.0011) and 15 hpf (*p* = 0.040), respectively. Interestingly, by 24 hpf, both *axin2* (1.20±0.08-fold increase, *p* = 0.004) and *lef1* (1.99±0.28-fold increase, *p* = 0.022) expression levels were significantly higher in *rbc3a*-deficient embryos than in controls. *rbc3a*-MO1 injection also significantly increased *mitfa* expression at 24 hpf (2.29±0.10-fold increase, *p*<0.001).

To confirm these changes in Wnt signaling specifically in NC, we examined the subcellular localization of Bcat in response to *rbc3a*-MO1 injection in NC cells using a Bcat antibody in *sox10:lyn-gfp* transgenics. At 11 hpf, prior to NC migration, Bcat co-localized significantly more with GFP+ NC cell membranes in *rbc3a*-MO1–injected embryos compared to controls, where the protein localized more to nuclei based on colocalization with DAPI (Figure 5B–C″, Figure S9A, *n* = 13 embryos, *p*<0.001). This difference was also seen in V0a1 knockdown embryos at 11 hpf (*p* = 0.038) (Figure S10B–B″). At 14 hpf, there was not a significant difference in the nuclear localization of Bcat in *rbc3a-*MO1–injected and wild-type NC cells. By 24 hpf, when most NC cells have already migrated in wild-type embryos, the situation appeared to be reversed—Bcat was largely at the membrane in control NC cells and significantly more nuclear in NC cells in *rbc3a*-MO1–injected embryos (Figure 5D–E″, Figure S9A, *n* = 15 embryos, *p*<0.001) as well as *v0a1*-deficient embryos (Figure S10D–E, *p* = 0.014). These results suggest that in *rbc3a*-deficient NC cells, Wnt signaling is initially reduced prior to their EMT and later elevated once cells have begun to migrate.

### Rbc3a Knockdown Disrupts Subcellular Localization of Fz7

Endocytosis of Fz receptors is required for their turnover and degradation, as well as in some cases to concentrate Wnt-bound Fz receptors in so-called signalosomes, which enhance Wnt signaling [20],[31]. To examine if Rbc3a and/or V0a1 depletion alters the endocytosis and/or subcellular localization of Wnt receptors, mRNA encoding an N-terminal tagged zebrafish *frizzled7b* (*fz7-yfp*, [32]) was injected into *Tg(7.2 kb-sox10:lyn-tdtomato)* transgenics, controls, and embryos injected with *rbc3a*-MO1 and later detected using an anti-GFP antibody (Figure 5F–J). In contrast to controls, Fz7-YFP was significantly more abundant at the membranes of NC cells in *rbc3a-*MO1–injected embryos, particularly in dorsal aggregates of unmigrated cells at 20 hpf (Figure 5I,I′, Figure S9B, *n* = 7 embryos, *p*<0.001). Intensity measurements of YFP staining at the membrane and cytosol of NC cells showed a significant increase in the amount of Fz-YFP localized to the membrane in *rbc3a*-MO1–injected embryos at 20 hpf compared to wild-type (Figure S9B, *p*<0.001, *n* = 34 cells). This suggests that disruption of *rbc3a* prevents proper degradation and turnover of Fz7 receptors in NC cells, which could partially account for the elevated Wnt signaling in these cells.

### Rbc3a Depletion Alters Cell–Cell Adhesion Molecule Expression in NC

**Figure 6:**
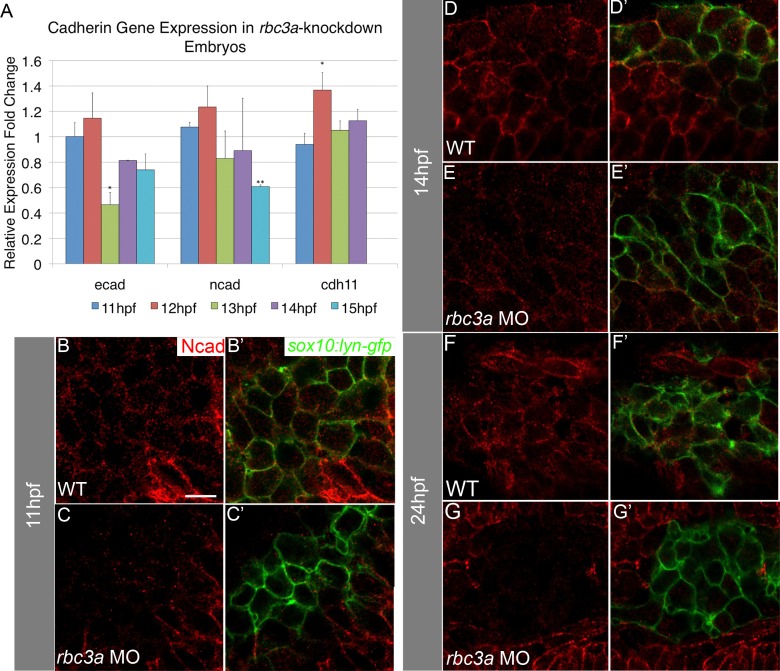
Rbc3a knockdown reduces expression of cadherins in NC cells. (A) Quantitative real-time PCR for *ecad*, *ncad*, and *cdh11* at four timepoints during the onset of NC migration in *rbc3a*-MO1–injected embryos. *ecad* expression is significantly down-regulated at 13 hpf and remains low. *ncad* expression is also significantly reduced by 15 hpf. In contrast, *cdh11* expression increases at 12 hpf. Error bars represent triplicate experiments ± SEM. (B–G′) Confocal images of immunohistochemical staining for Ncad (red) in *sox10:lyn-gfp* transgenics (green) at 11 hpf (B–C′), 14 hpf (D–E′), and 24 hpf (F–G′). Ncad levels are reduced at the membranes of GFP+ cells by 14 hpf in *rbc3a*-MO1–injected embryos and almost completely absent in the NC of *rbc3a*-MO1–injected embryos by 24 hpf, while still present at the membrane in surrounding cells. * *p*<0.05, ** *p*<0.01. Scale bar, 10 µm.

Regulation of cell–cell adhesion molecules, such as cadherins, is necessary for proper EMT and migration of NC cells. Snai2 directly represses E-cadherin (Ecad) [33] and regulates the transition from *ecad* to *ncad* expression [34]. During EMT and early migration, *ecad* is down-regulated and *ncad* up-regulated in response to canonical Wnt signaling in NC cells as they delaminate and become motile. With qPCR analysis of mRNA levels (Figure 6A) at 12–13 hpf in *rbc3a*-MO1–injected embryos, we found that *ecad* expression was significantly reduced (*p*<0.001) compared with controls and by 15 hpf *ncad* expression was significantly reduced (*p* = 0.002). Cadherin-11 (Cdh11) is a canonical Wnt target expressed throughout NC migration but must be tightly regulated as its overexpression prevents NC migration [35]. We found that *cdh11* expression was up-regulated in *rbc3a-*MO1–injected embryos (Figure 6A, *p* = 0.028). The timing of reduction in *ncad* expression and up-regulation of *cdh11* in *rbc3a*-MO1–injected embryos correlates with the initial decreases in canonical Wnt targets (Figure 5A). To determine if Rbc3a also regulates the subcellular localization of cadherins, we performed immunohistochemical staining for Ncad. Similar to our qPCR results, Ncad abundance at NC cell membranes was not significantly different than wild-type embryos at 11 hpf (Figure 6B–C′, Figure S9C). The reduction in abundance of Ncad was significant by 14 hpf in NC but not adjacent cells (Figure 6D–E′, *n* = 12 embryos, *p*<0.001) and remained reduced at 24 hpf in NC cells of *rbc3a*-MO1–injected embryos (Figure 6F–G′, *n* = 11 embryos, *p*<0.001). Thus, in addition to Wnt signaling defects, *rbc3a*-deficient NC cells also display changes in *cadherin* expression levels during EMT and early migration, possibly contributing to their inability to delaminate from the neural tube and migrate.

## Discussion

NC cells in embryos deficient in either *rbc3a* or *v0a1* accumulate large EEA1-positive, acidified endosomes and decreased late endosomes/lysosomes, which we interpret as a block in endosomal processing. Previous work has shown that various V0a isoforms confer targeting of v-ATPase to specific subcellular compartments, while binding partners of V0a such as Rbc3a and its orthologues regulate assembly and/or activity of v-ATPase. Endosomal processing defects in *rbc3a*/*v0a1*-deficient NC cells resemble those seen in Drosophila photoreceptors, zebrafish microglia, and mouse embryonic fibroblasts lacking V0a1, all of which develop large acidified endosomal aggregates [27],[28],[36]. In other cell types, however, such as fly ovarian follicle or wing disc cells, or mammalian osteoblasts [24]–[26], Rbc3a and V0a1 control organelle acidification by regulating v-ATPase activity. Although acidification defects were not observed in NC cells of *rbc3a* or *v0a1*-deficient zebrafish embryos, V0a1 does regulate intracellular acidification of zebrafish hair cells of the lateral line [26]. Thus, deficiency of V0a1 in the same organism (zebrafish) causes different phenotypes (i.e., endosomal processing versus acidification defects) depending on the cell type examined.

Such cell-type specificity in V0a subunits is not unprecedented. Fly ovaries express high levels of the v0a2 subunit homolog and low levels of v0a1 [37]. The v0a3 subunit is expressed specifically in mammalian osteoclasts [38]. Both V0a1 and V0a2 isoforms regulate both endocytosis and storage of neurotransmitter-containing vesicles, rather than just exocytosis [39]. Rbc3a resides in synaptic vesicles in mammalian neurons [40] and drives co-localization of V0a1 and V1a1 subunits in zebrafish hair cells [26]. Thus, V0a1/2 subunits can control vesicle uptake and storage in addition to their better-known roles in lysosomal acidification and may do so together with Rbc3a in many other cell types.

**Figure 7:**
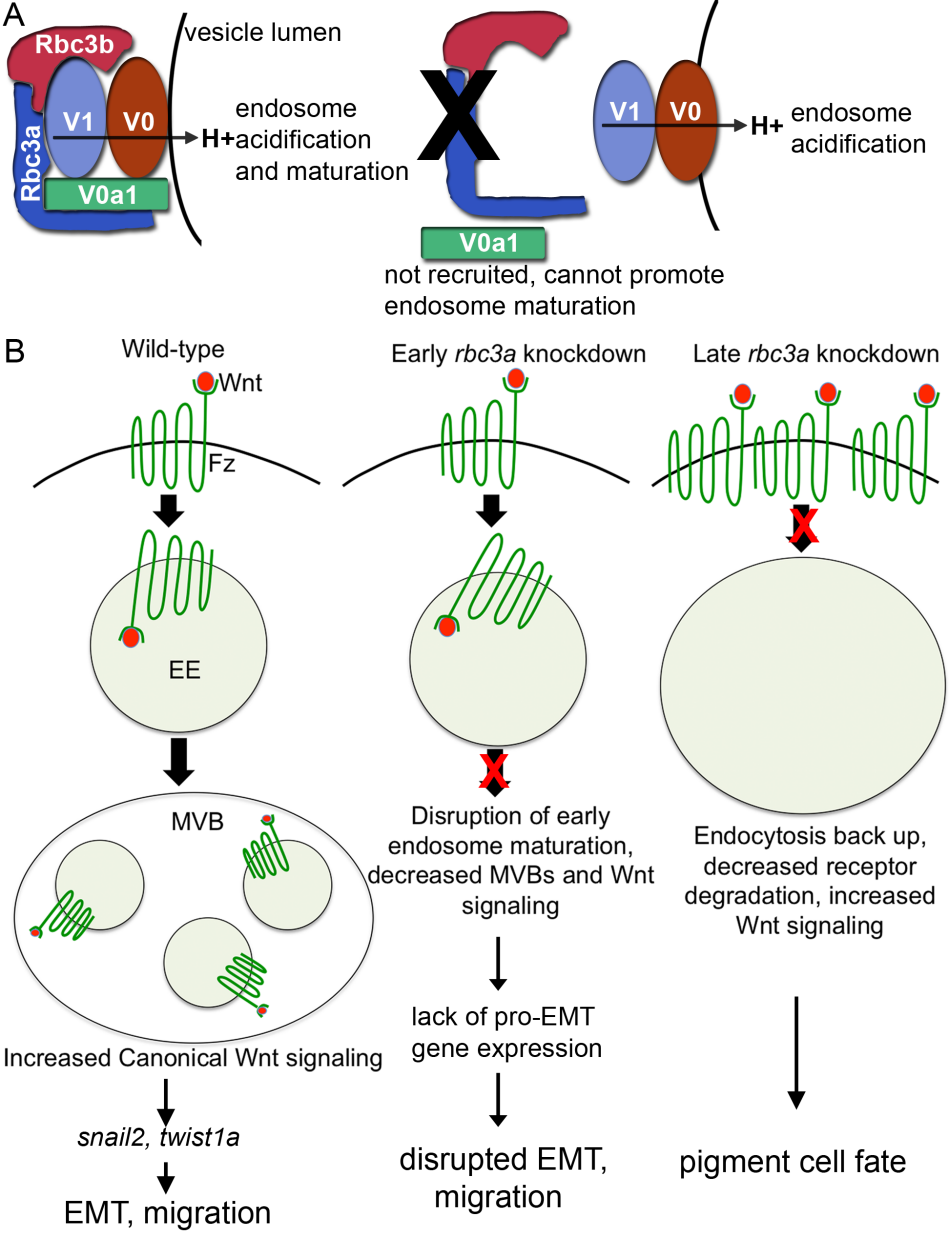
Model for Rbc3a and V0a1 functions in NC cells. (A) Rbc3a promotes association of a specific v0a1 isoform to early endosomes. In the absence of Rbc3a, endosomes acidify, but do not mature. (B) Rbc3a controls maturation of early endosomes (EE) containing Fz-Wnt complexes to contribute to multivesicular bodies (MVBs), which promote canonical Wnt signaling and EMT. In the absence of Rbc3a (or v0a1), canonical Wnt signaling decreases early (∼11 hpf) and increases later (∼20 hpf). Defects in EE maturation disrupt Fz-Wnt recycling and degradation, producing high levels of Fz-Wnt complexes at the cell membrane and aberrantly high levels of canonical Wnt signaling, which drives NC cells to a pigment progenitor fate.

One possible explanation for the ability of endosomes to acidify in certain cell types deficient in Rbc3a and/or V0a1 likely relates to differences in binding partners for V0a1 and/or multiple isoforms of V0a that may exist in various cell types. For example, Drosophila V0a1 binds to a syntaxin necessary for endosomal membrane fusion, which likely explains why Drosophila photoreceptors deficient in V0a1 exhibit an early endosomal processing defect [28]. In addition, elegant studies in Drosophila have dissociated the regulation of endosomal processing from that of v-ATPase-activity in V0a1 [28]. V0a1 is not required for lysosomal acidification in mouse embryonic fibroblasts or rat PC12 cells [36]. Our results suggest that Rbc3a and V0a1 regulate endosomal fusion but not acidification by v-ATPase in NC cells (Figure 7A).

Our results further suggest that abnormal endosomal maturation alters Wnt signaling in NC cells lacking Rbc3a and V0a1. Canonical Wnt signaling requires endocytosis, and intracellular v-ATPase–mediated acidification is necessary for phosphorylation of the Wnt receptor LRP6 [20]. Regulation of endocytosis and v-ATPase activity by Rbc3a/b also plays a critical role in Notch signaling [24],[25]. Mounting evidence suggests that signal transduction via multiple cell surface receptors must occur in endosomal signaling protein complexes or “signalosomes” [31],[41]. Signalosomes allow coordinated regulation of receptor signaling, recycling, and/or degradation in a regulated microenvironment. We hypothesize that a reduction of Rbc3a and V0a1 disrupts signalosome function and interferes with Wnt signaling in NC cells. During early NC development, we observed increased cell surface staining of Bcat and reduced expression of multiple canonical Wnt target genes. During later stages of development, however, we observed a paradoxical increase in Wnt signaling in midline NC aggregates that failed to migrate. These nonmigrating midline NC aggregates showed increased cell surface Fz staining, increased nuclear Bcat staining, up-regulation of Wnt target genes, and became specified as pigment cells, which is known to be driven by canonical Wnt signaling [16]. Impaired lysosomal function and a build-up of endosomes can increase Wnt signaling [42]. Other Fz receptors and components of canonical Wnt signaling known to accumulate in endosomal bodies, such as GSK3B [31], may also contribute to this elevation in Wnt signaling. The early reduction and later increase in Wnt signaling in NC cells caused by Rbc3a and V0a1 deficiency may be secondary to differential expression of other signalosome components or other mechanisms of Wnt signaling that occur at different stages of embryogenesis (Figure 7B).

Changes in Wnt signaling, as well as endocytosis and Fz receptor localization, in response to Rbc3a knockdown appear to be NC-specific. Rbc3a-deficient NC cells display both reduced migration velocity and persistent directionality. Additionally, transplanted Rbc3a-deficient NC cells fail to migrate in a wild-type host. These observations and the specific expression of *rbc3a* in premigratory NC cells suggest a cell autonomous role for Rbc3a in NC development.

Both canonical (Bcat/Tcf-dependent) and noncanonical (PCP, Ca^2+^-dependent) Wnt signaling plays important roles in NC development [43], but several lines of evidence suggest that the main requirements for Rbc3a are in canonical signaling. First, *rbc3a* expression is highly enriched in NC cells prior to migration, when they require canonical Wnt signaling for induction and EMT, but not during later stages of migration, when noncanonical signaling has been implicated [44]. Second, loss of Rbc3a leads to specific defects in pigment cells, which are induced by canonical Wnt signaling [45]. Third, the timing of changes in expression of Wnt target genes, nuclear localization of Bcat, and membrane localization of Fz7b correlate with stages in which canonical signaling predominates, though Fz7b has been implicated in both canonical and noncanonical Wnt pathway activation [43],[46].

Canonical Wnt signaling plays a major role in NC induction [30],[47],[48], but we have not detected defects in NC induction in *rbc3a*-MO1–injected or mutant embryos. Rbc3a may only affect certain Wnt signaling pathways or Wnt family members, leading to specific downstream effects in migration but not NC induction. Alternatively, maternal *rbc3a* mRNA or protein may be sufficient to allow induction but not later stages of NC development.

We demonstrate that Rbc3a and V0a1 play important and novel roles in NC EMT and cell migration. Canonical Wnt signaling promotes NC EMT and delamination [10],[14] by up-regulating expression of pro-EMT transcription factors such as Gbx2 [49], Snail2/Slug [12], and Twist1 [13]. Our data indicate that Rbc3a and V0a1 are required for this Wnt response and suggest that they coordinate both cell signaling and cell adhesion during migration. Rbc3a and V0a1 knockdown reduce Bcat nuclear localization in premigratory NC as well as expression of pro-EMT, NC-specific Wnt targets. Snail1/2 and Twist1 promote EMT, in part by regulating expression of cell adhesion molecules, including Ecad [33] and Ncad levels [13]. We show that in addition to reductions in *snail2* and *twist1a* expression, Rbc3a deficiency reduces *ncad* levels at the onset of NC migration. It also increases expression of *cdh11*, which normally must be down-regulated for proper NC EMT and delamination [6],[35]. Because of the close association between cadherins and Bcat, these differences in cadherin expression could be downstream of defects in Wnt signaling and pro-EMT target gene expression or could reflect defects in trafficking of cadherins directly. Either could explain the failure of *rbc3a*-deficient NC cells to undergo EMT, delaminate, and migrate. However, our results suggest that the reduction in canonical Wnt signaling comes first. We detect reductions in expression of canonical Wnt target genes by qPCR and *in situ* hybridization 2–3 h before changes in Ncad and other cadherins' (Ecad, Cdh11) expression in *rbc3a*-MO1–injected embryos. In addition, nuclear localization of Bcat protein is reduced relative to its levels in membrane/cytoplasm in *rbc3a*-MO1–injected embryos at 11 hpf, prior to any differences in abundance or membrane versus cytoplasmic localization of Ncad protein.

Cells undergoing EMT and migration require the regulated function of several intertwined cellular processes: endocytosis, signal transduction, and cell adhesion molecule localization. Rbc3a and V0a1 provide a novel link between all of these processes in NC development. Although previous work has shown the importance of Rbc3a in Notch signaling, we propose a novel role for Rbc3a in Wnt signaling in NC cells, as well as a more general role in EMT and cell migration through the recruitment of V0a1. The yeast orthologues of Rbc3a and Rbc3b simply promote the association of the V0 and V1 units of the v-ATPase and its function in acidification [50]. In multicellular organisms, Rbc3a may have gained a role driving the association of specific v-ATPase subunit isoforms and other adaptor proteins in specialized cell types and specific cellular transitions. Rbc3a is expressed in multiple migratory cell types in the zebrafish and other species, and Rbc3a's potential role in EMT/migration may serve as a new avenue of developmental biological investigation as well as a therapeutic target in cancer metastasis.

## Materials and Methods

### Zebrafish

Zebrafish embryos were obtained from natural breeding, raised, and staged as described previously [51]. *Stardust* mutants heterozygous for the *rbc3a^Q850X^* allele were obtained from the Nicolson laboratory [26]. Transgenic lines containing a 7.2 kb region of the *sox10* promoter driving expression of cytoplasmic *gfp* [29] or plasma-membrane localized *lyn-gfp* and *lyn-tdTomato* were used to visualize NC cells [*Tg(7.2 kb-sox10:gfp)*, *Tg(7.2 kb-sox10:lyn-gfp)*, and *Tg(7.2 kb-sox10:lyn-tdTomato)*, respectively].The Wnt reporter line, *Tg(7xTCF-Xla.Siam:GFP)^ia^*, was obtained from the Dorsky laboratory [45].

### Cloning of Rbc3a

To obtain full-length *rbc3a*, portions of the predicted ORF were subcloned using the TOPO TA Cloning Kit (Invitrogen) and sequenced. The full-length sequence was assembled with Lasergene SeqMan, and *rbc3a* with 5′ KpnI and 3′ NotI restriction sites was amplified from a 24 hpf cDNA library using *rbc3a KpnI F* and *rbc3a NotI R* primers, digested, and cloned into the KpnI and NotI sites of the Gateway #237 pME-MCS vector and then recombined with the p5E-CMV/Sp6 and p3E-MTpA vectors into the pDestination vector #393 Tol2CG.

### Microinjections

Antisense morpholino oligonucleotides (MOs) targeting the *rbc3a* 5′ untranslated region (*rbc3a*-MO1), translation start site (*rbc3a*-MO2), and the previously described *atp6v0a1* intron/exon boundary (*v0a1*-MO [27]) were purchased from Gene Tools and dissolved in 1× Danieau buffer for injection. For MO experiments, 1–3 ng of *rbc3a-*MO1/embryo, 5 ng of *rbc3a*-MO2/embryo, or 4 ng of *v0a1-*MO/embryo was injected into one- to four-cell-stage embryos along with 1 ng *p53*-MO/embryo to inhibit nonspecific cell death. Unless otherwise noted, all Rbc3a knockdown experiments were performed with *rbc3a*-MO1. See Table S2 for MO sequences. For RNA injection experiments, the full-length ORFs of *rbc3a* in Tol2CG or zebrafish *frizzled7b*-*YFP* (*fz7-yfp*) pCS2+ construct [32] were translated using mMessage mMachine kit (Ambion) and injected at the one-cell stage. A pCMVSPORT6 DNA vector containing a shortened ORF of *Xenopus rbc3a* (Open Biosystems, Accession No. BC127555) was injected at the one-cell stage.

### Genotyping of *rbc3a^Q850X^* Mutants

Mutants were incrossed and embryos were sorted by morphological phenotype. Genomic DNA was isolated from individual embryos, amplified (forward – TGTTCTGTTTGTGTTGCTCAG, reverse – CCTTCTCCAGAGGGAAAACT) and sequenced to identify *rbc3a*−/− mutants.

### RNA *in Situ* Hybridization and Immunohistochemistry

Whole mount RNA *in situ* hybridization was performed as previously described [52]. Probes used for NC cell analysis included *sox10*, *mitfa*, *gch*, *dlx2a*, *snai2*, *gbx2*, *axin2*, *twist1a*, and *foxd3* [53]. *rbc3a* probe template was amplified from 24 hpf cDNA with *rbc3a probe F* and *R* primers (see Table S3) and probe synthesized with T7 RNA polymerase and hybridized as previously described [54].

Zebrafish N-cadherin (Ncad) polyclonal antibody was generated by GeneTex in rabbit using the region between amino acids 440 and 729 (Accession No. AAI33732). Staining conditions and antibodies used are summarized in Table S4. Briefly, embryos were washed in PBT (phosphate buffer saline with 0.1% Triton-X100 and 1% DMSO) after overnight fixation in 4% paraformaldehyde and blocked with 10% goat serum for at least 1 h at room temperature. Incubations with primary were performed overnight at 4°C and secondary antibody incubations were performed for 2 h at RT with extensive washing in PBT in between both incubations.

Vesicle acidification was measured by incubating embryos with 10 µM Lysotracker Red DND-99 dye (Invitrogen) in embryo medium for 1 h, washed several times, and imaged.

### Phenotypic Scoring

The NC migration phenotype in live *sox10:gfp* embryos was scored by the presence of ≥3 aggregates of multiple cells in the dorsal area from the MHB to the posterior edge of the otic vesicle by 20 hpf in triplicate experiments and analyzed by Chi-squared tests. Counts of dorsal midline NC cells were scored on fixed *sox10:gfp* embryos stained with DAPI in the dorsal region between the MHB and otic vesicle. Particle counts of intracellular vesicles stained for EEA1, LAMP1, or Lysotracker Red were made by drawing regions of interest around *sox:gfp*+ cells in ImageJ of confocal images and processed with the Particle Analyzer function using a lower threshold of 2×2 pixels on *n*≥7 embryos. Fz-YFP, Ncad, and Bcat subcellular localization was quantified by drawing regions of interest with identical areas at the membrane, within the cytosol, and/or nucleus of NC cells in confocal image slices of 24 hpf *sox10:lyn-tdTomato–* or *sox10:lyn-gfp*–injected embryos. The mean intensity of YFP, Ncad, and Bcat at the membrane, cytosol, and/or nucleus in individual cells was analyzed in ImageJ to quantify the intensity of staininig in different subcellular locations.

### Cell Transplantation

Wild-type cells and cells injected with 2 ng *rbc3a*-MO1 were grafted at gastrula stages from *sox10:lyn-tdtomato* transgenic donors into nontransgenic hosts as previously described [9]. Embryos were selected based on NC-specific *sox10:lyn-tdtomato* expression and photographed at 24 hpf. NC migration defects were scored by counting aggregates of ≥3 cells in the dorsal midline from the MHB to the posterior edge of the otic vesicle at 24 hpf. Results were statistically analyzed by Chi-squared. Transplantation experiments were not performed with *rbc3a*-MO2–injected embryos.

### Confocal Imaging and Movies

Confocal images were taken using an Olympus Fluoview FV1000 with a 60× oil-immersion lense and processed in ImageJ. To analyze NC cell migration, NC cells were labeled by the *sox10:gfp* line. Transgenic embryos were manually dechorionated, anesthetized with ethyl-m-aminobenzoate methane sulfanate, and mounted in 1% agarose in embryo medium on a coverslip and imaged. For time-lapse imaging, embryos were imaged on a Nikon Eclipse Ti spinning disk microscope equipped with a 40×/1.15 WI Apo LWD objective. Approximately 40 µm z-stacks were captured at 0.5 µm intervals every 2 min for 4 h and edited in ImageJ at 7 frames/s beginning at ∼13 hpf for movies.

Cell migration was assayed at the onset of cranial NC migration (∼12–15 hpf) by tracking cell movements in ImageJ with Manual Tracking. Average migration speed was calculated as total distance traveled over time and directionality assayed by calculating persistence (defined as distance between initial and final positions over total distance traveled) as previously described [55]. Statistical significance was assessed by two-tailed Student's *t* test, α = 0.05.

### Quantitative Real-Time PCR

Total RNA was isolated from embryos using Trizol reagent (Gibco/BRL). First-strand cDNA synthesis was performed on 1 µg of total RNA using oligo dT primers and Superscript III reverse transcriptase (Invitrogen). Quantitative real-time RT-PCR (qPCR) was performed using SYBR Green 1 Master PCR mix (Roche) in a LightCycler 480 System (Roche) with biological triplicates using the qPCR primer sets in Table S3.

### Statistics

Unless otherwise noted, data were compared with one-way ANOVA with a post hoc Tukey test to compare multiple means with an α = 0.05.

## Supporting Information

Figure S1 Rbc3a loss-of-function phenotype at 72 hpf. (A, B, D, F) Increasing amounts of *rbc3a-*MO1 (1–3 ng/embryo) leads to cardiac edema, reduced melanocytes, and shortened, curved tails in larvae at 72 hpf. (C, E, G) *rbc3a* mutant larvae show similar phenotypes. Scale bar, 200 µm.
(TIF)

Figure S2 Rescue of *rbc3a*-MO1–injected embryos with full-length *rbc3a* mRNA. (A–C) Fluorescent images of live *sox10:gfp* transgenics, dorsal views, anterior to the left: (A) wild-type (WT), (B) *rbc3a*-MO1 injected, and (C) co-injected with full length *rbc3a* mRNA. (D) Number of GFP+ cells aggregated at the dorsal midilne at 24 hpf and located between the otic vesicle and MHB along the A–P axis. Injection of 100 pg *rbc3a* mRNA significantly rescued the number of GFP+ cells from 27.4±2.7 in *rbc3a*-MO1–injected embryos (*n* = 8) to 10.8±3.0 cells in mRNA+MO injected embryos (*p*<0.001, *n* = 8). Injection of 100 pg mRNA alone (*n* = 8) had no effect on the number of midline GFP+ cells compared to 2.3±1.7 in wild-type embryos (*n* = 9). Error bars represent ± SEM. Scale bar, 100 µm.
(TIF)

Figure S3 Injection of *rbc3a*-MO1 or -MO2 produces similar NC defects. (A–F) Live *sox10:gfp* embryos at 24 hpf, dorsal (A–C) and lateral (D–F) views, anterior to the left. Compared to wild-type embryos (A, D), embryos injected with either *rbc3a-*MO1 (B, E) or *rbc3a*-MO2 (C, F) display similar *sox10:gfp*+ dorsal midline cell aggregates (white arrowheads).
(TIF)

Figure S4 Injection of a 3′-truncated Xenopus *rbc3a* construct phenocopies Rbc3a loss of function. Fluorescent images of live *sox10:gfp* transgenics, lateral views, anterior to the left. (A, B) Injection of 1 ng/embryo of *rbc3a*-MO1 caused GFP+ cells to aggregate at the dorsal midline (arrowheads) by 24 hpf (41%, *n* = 7/17). (C) Injection of 100 pg/embryo of Xenopus *rbc3a* mRNA lacking 2.2 kb of the 3′ end of the ORF caused similar GFP+ dorsal aggregates (83%, *n* = 15/18). (D) Co-injection of 50 pg/embryo of truncated *xrbc3a* mRNA with 1 ng/embryo of *rbc3a*-MO1 increased the number and severity of embryos with GFP+ dorsal aggregates (79%, *n* = 11/14), with some embryos exhibiting a continuous strip of GFP+ cells all along the dorsal midline (36%, *n* = 5/14).
(TIF)

Figure S5 Reduced NC cell motility in *rbc3a*-MO1–injected embryos. Individual frames from confocal time-lapsed movies of wild-type (A–E) and *rbc3a*-deficient (G–K) embryos from 13 hpf onwards in 20-min intervals. (F, L) Cell trajectories over 2 h of the corresponding cells in (A, F, and G–K). Wild-type NC cells (F) display stereotypical rapid, directed movement laterally and anteriorly, while many NC cells in *rbc3a*-MO1–injected embryos (L) adhere to each other and fail to migrate with other NC cells. (M) Average NC cell migration speed and (N) persistence of directionality (measured as the total displacement from the starting position of a cell over the total path length) at the onset of migration in wild-type and *rbc3a*-MO1–injected embryos (MO). Compared to wild-types, *rbc3a*-MO1 injection led to significantly reduced migration speed (*p* = 0.0028, 1.58±0.32 and 1.09±0.15 µm/min, respectively) and persistence (*p* = 0.015, 0.73±0.17 and 0.38±0.28, respectively). Error bars represent ±SEM. * *p*<0.05, ** *p*<0.01.
(TIF)

Figure S6 Enlarged early endosomes grow and accumulate over time in NC cells of *rbc3a*-MO1–injected embryos. Confocal images of *sox10:gfp*+ NC cells (bottom row, green) double labeled with anti-EEA1 (A–F, red) and anti-Rab11a (A′–F′, blue), which marks late endosomes. (A″–F″) Merged images of EEA1, Rab11a, and *sox10:gfp* fluorescence. EEA1+ vesicles increase in number and size in *rbc3a*-MO1–injected embryos from 14–20 hpf compared to wild-type controls, while Rab11a+ vesicles show no change. Scale bars, 10 µm.
(TIF)

Figure S7 Enlarged early endosomes acidify in NC cells migrating into the pharyngeal arches of *rbc3a*-MO1–injected embryos. (A–B″) Lateral view of live *sox10:lyn-gfp* transgenic embryos showing pharyngeal arches stained with Lysotracker-Red. NC cells in *rbc3a*-MO1–injected embryos contain larger and more Lysotracker-positive intracellular vesicles (B′, B″) compared to controls. Scale bar, 10 µm.
(TIF)

Figure S8 Changes in downstream Wnt target gene expression. (A–H) *In situ* hybridization for Wnt target gene expression in wild-type and *rbc3a-*MO1–injected embryos. Dorsal views (A–F), lateral views (G, H). Several genes display reduced expression in the NC (arrowheads) in *rbc3a*-MO1–injected embryos from 10–12 hpf including *gbx2* (A, B), *snail2* (C, D), *twist1a* (E, F), and *axin2* (G, H). Scale bar, 100 µm. (I) *axin2*, *nmyc*, and *lef1* show decreased expression by 13 hpf in *rbc3a*-MO1–injected embryos. Error bars represent triplicate experiments ± SEM. * *p*<0.05.
(TIF)

Figure S9 Cell autonomous effects of Rbc3a knockdown. (A–C) Quantification of subcellular localization of (A) Bcat, (B) Fz7b-YFP, and (C) Ncad in NC and non-NC cells. ** *p*<0.01, *** *p*<0.001, N.S., not significant. (D) Wild-type host embryo at 24 hpf with *sox10:lyn-tdtomato* cells (red) transplanted from a *rbc3a*-MO1–injected donor, dorsal view. A subset of *rbc3a*-deficient donor cells formed dorsal midline aggregates (white arrowheads), but many other cells migrated properly into the pharyngeal arches (asterisk). Mhb, mindbrain-hindbrain boundary. Scale bar, 100 µm.
(TIF)

Figure S10 V0a1 knockdown disrupts nuclear localization of Bcat in NC. (A–D″) Confocal images of *sox10:gfp* (green) embryos stained with DAPI (blue) and an anti-Bcat antibody (red) in uninjected controls (A–A″, C–C″) and embryos injected with *v0a1*-MO at 11 hpf (B–B″) and 24 hpf (D–D″). V0a1 knockdown reduces levels of B-cat in NC cell nuclei at 11 hpf (B–B″) and nuclear Bcat at 24 hpf (D–D″). Scale bars, 10 µm. (E) Quantification of the ratio of Bcat localization between NC cell membranes and nuclei indicates a significant decrease in nuclear localization in *rbc3a*-MO1–injected embryos at 11 hpf but a significant increase in nuclear localization at 24 hpf. Errors bars respresent ± SEM, * *p*<0.05.
(TIF)

Movie S1 Time lapse movie of NC migration in a *sox10:gfp* control. Dorsal view, anterior to the left, showing NC cell migration starting at ∼13 hpf.
(AVI)

Movie S2 Time lapse movie of NC migration in a *rbc3a-*MO1–injected *sox10:gfp* embryo. Dorsal view, anterior to the left, showing NC cell migration starting at ∼13 hpf in a *rbc3a*-MO1–injected embryo.
(AVI)

Table S1 Similar dorsal midline aggregates of NC cells following *rbc3a*-MO2 injection further confirm morpholino specificity. Both control and *rbc3a* mRNA-injected embryos show no NC defects. In contrast, *rbc3a*-MO2–injected embryos display dorsal midline NC cells (85.3%) compared to control embryos (*p*≪0.0001). Co-injection of *rbc3a* mRNA with *rbc3a*-MO2 partially rescues these NC defects (47.3%; *p*≪0.0001).
(DOCX)

Table S2 Antisense MO design.
(DOCX)

Table S3 Primer sequences: 5′-3′ sequences of qPCR and probe synthesis primers.
(DOCX)

Table S4 Antibody staining sources and conditions.
(DOCX)

## Abbreviations

Bcat: B-catenin
Cdh: Cadherin
DAPI: 4′,6-diamidino-2-phenylindole
Ecad: E-cadherin
EEA1: Early Endosome Antigen 1
EMT: epithelial-mesenchymal transition
Fz7: Frizzled-7
GSK3B: Glycogen synthase kinase 3 beta
hpf: hours postfertilization
LAMP1: Lysosomal Associated Membrane Protein 1
LRP5/6: low-density lipoprotein receptor-related protein 5/6
MO: morpholino oligonucleotide
NC: neural crest
Ncad: N-cadherin
Rbc3a: Rabconnectin-3a
UTR: untranslated region
v-ATPase: vacuolar ATPase
YFP: yellow fluorescent protein

## References

[1] Hay ED (1995) An overview of epithelio-mesenchymal transformation. Acta Anat (Basel) 154: 8–20. 8714286 10.1159/000147748

[2] Thiery JP (2002) Epithelial-mesenchymal transitions in tumour progression. Nat Rev Cancer 2: 442–454. 12189386 10.1038/nrc822

[3] Kerosuo L, Bronner-Fraser M (2012) What is bad in cancer is good in the embryo: importance of EMT in neural crest. Semin Cell Dev Biol 23: 320–332. 22430756 10.1016/j.semcdb.2012.03.010 PMC3345076

[4] Le Douarin NM, Creuzet S, Couly G, Dupin E (2004) Neural crest cell plasticity and its limits. Development 131: 4637–4650. 15358668 10.1242/dev.01350

[5] Stuhlmiller TJ, Garcia-Castro MI (2012) Current perspectives of the signaling pathways directing neural crest induction. Cell Mol Life Sci 69: 3715–3737. 22547091 10.1007/s00018-012-0991-8 PMC3478512

[6] Nakagawa S, Takeichi M (1995) Neural crest cell-cell adhesion controlled by sequential and subpopulation-specific expression of novel cadherins. Development 121: 1321–1332. 7540531 10.1242/dev.121.5.1321

[7] Nakagawa S, Takeichi M (1998) Neural crest emigration from the neural tube depends on regulated cadherin expression. Development 125: 2963–2971. 9655818 10.1242/dev.125.15.2963

[8] Monier-Gavelle F, Duband JL (1995) Control of N-cadherin-mediated intercellular adhesion in migrating neural crest. J Cell Sci 108 (Pt 12) 3839–3853. 8719890 10.1242/jcs.108.12.3839

[9] Piloto S, Schilling TF (2010) Ovo1 links Wnt signaling with N-cadherin localization during neural crest migration. Development 137: 1981–1990. 20463035 10.1242/dev.048439 PMC2875841

[10] de Melker AA, Desban N, Duband JL (2004) Cellular localization and signaling activity of beta-catenin in migrating neural crest cells. Dev Dyn 230: 708–726. 15254905 10.1002/dvdy.20091

[11] Katoh M (2006) Cross-talk of WNT and FGF signaling pathways at GSK3beta to regulate beta-catenin. Cancer Biol Ther 5: 1059–1064. 16940750 10.4161/cbt.5.9.3151

[12] Wu ZQ, Li XY, Hu CY, Ford M, Kleer CG, et al (2012) Canonical Wnt signaling regulates Slug activity and links epithelial-mesenchymal transition with epigenetic Breast Cancer 1, Early Onset (BRCA1) repression. Proc Natl Acad Sci U S A 109: 16654–16659. 23011797 10.1073/pnas.1205822109 PMC3478591

[13] Howe LR, Watanabe O, Leonard J, Brown AM (2003) Twist is up-regulated in response to Wnt1 and inhibits mouse mammary cell differentiation. Cancer Res 63: 1906–1913. 12702582

[14] Burstyn-Cohen T, Stanleigh J, Sela-Donenfeld D, Kalcheim C (2004) Canonical Wnt activity regulates trunk neural crest delamination linking BMP/noggin signaling with G1/S transition. Development 131: 5327–5339. 15456730 10.1242/dev.01424

[15] LaBonne C, Bronner-Fraser M (2000) Snail-related transcriptional repressors are required in Xenopus for both the induction of the neural crest and its subsequent migration. Dev Biol 221: 195–205. 10772801 10.1006/dbio.2000.9609

[16] Dorsky RI, Moon RT, Raible DW (1998) Control of neural crest cell fate by the Wnt signalling pathway. Nature 396: 370–373. 9845073 10.1038/24620

[17] Hari L, Miescher I, Shakhova O, Suter U, Chin L, et al (2012) Temporal control of neural crest lineage generation by Wnt/beta-catenin. Development 139: 2107–2117. 22573620 10.1242/dev.073064

[18] Blitzer JT, Nusse R (2006) A critical role for endocytosis in Wnt signaling. BMC Cell Biol 7: 28. 16824228 10.1186/1471-2121-7-28 PMC1534015

[19] Bryja V, Cajanek L, Grahn A, Schulte G (2007) Inhibition of endocytosis blocks Wnt signalling to beta-catenin by promoting dishevelled degradation. Acta Physiol (Oxf) 190: 55–61. 17428233 10.1111/j.1365-201X.2007.01688.x

[20] Bilic J, Huang YL, Davidson G, Zimmermann T, Cruciat CM, et al (2007) Wnt induces LRP6 signalosomes and promotes dishevelled-dependent LRP6 phosphorylation. Science 316: 1619–1622. 17569865 10.1126/science.1137065

[21] Cruciat CM, Ohkawara B, Acebron SP, Karaulanov E, Reinhard C, et al (2010) Requirement of prorenin receptor and vacuolar H+-ATPase-mediated acidification for Wnt signaling. Science 327: 459–463. 20093472 10.1126/science.1179802

[22] Sakisaka T, Takai Y (2005) Purification and properties of rabconnectin-3. Methods Enzymol 403: 401–407. 16473606 10.1016/S0076-6879(05)03035-1

[23] Li KW, Chen N, Klemmer P, Koopmans F, Karupothula R, et al (2012) Identifying true protein complex constituents in interaction proteomics: the example of the DMXL2 protein complex. Proteomics 12: 2428–2432. 22707207 10.1002/pmic.201100675

[24] Yan Y, Denef N, Schupbach T (2009) The vacuolar proton pump, V-ATPase, is required for notch signaling and endosomal trafficking in Drosophila. Dev Cell 17: 387–402. 19758563 10.1016/j.devcel.2009.07.001 PMC2758249

[25] Sethi N, Yan Y, Quek D, Schupbach T, Kang Y (2010) Rabconnectin-3 is a functional regulator of mammalian Notch signaling. J Biol Chem 285: 34757–34764. 20810660 10.1074/jbc.M110.158634 PMC2966091

[26] Einhorn Z, Trapani JG, Liu Q, Nicolson T (2012) Rabconnectin3alpha promotes stable activity of the H+ pump on synaptic vesicles in hair cells. J Neurosci 32: 11144–11156. 22875945 10.1523/JNEUROSCI.1705-12.2012 PMC3428958

[27] Peri F, Nusslein-Volhard C (2008) Live imaging of neuronal degradation by microglia reveals a role for v0-ATPase a1 in phagosomal fusion in vivo. Cell 133: 916–927. 18510934 10.1016/j.cell.2008.04.037

[28] Williamson WR, Wang D, Haberman AS, Hiesinger PR (2010) A dual function of V0-ATPase a1 provides an endolysosomal degradation mechanism in Drosophila melanogaster photoreceptors. J Cell Biol 189: 885–899. 20513768 10.1083/jcb.201003062 PMC2878941

[29] Hoffman TL, Javier AL, Campeau SA, Knight RD, Schilling TF (2007) Tfap2 transcription factors in zebrafish neural crest development and ectodermal evolution. J Exp Zool B Mol Dev Evol 308: 679–691. 17724731 10.1002/jez.b.21189

[30] Garcia-Castro MI, Marcelle C, Bronner-Fraser M (2002) Ectodermal Wnt function as a neural crest inducer. Science 297: 848–851. 12161657 10.1126/science.1070824

[31] Taelman VF, Dobrowolski R, Plouhinec JL, Fuentealba LC, Vorwald PP, et al (2010) Wnt signaling requires sequestration of glycogen synthase kinase 3 inside multivesicular endosomes. Cell 143: 1136–1148. 21183076 10.1016/j.cell.2010.11.034 PMC3022472

[32] Witzel S, Zimyanin V, Carreira-Barbosa F, Tada M, Heisenberg CP (2006) Wnt11 controls cell contact persistence by local accumulation of Frizzled 7 at the plasma membrane. J Cell Biol 175: 791–802. 17130287 10.1083/jcb.200606017 PMC2064678

[33] Cano A, Perez-Moreno MA, Rodrigo I, Locascio A, Blanco MJ, et al (2000) The transcription factor snail controls epithelial-mesenchymal transitions by repressing E-cadherin expression. Nat Cell Biol 2: 76–83. 10655586 10.1038/35000025

[34] Hao L, Ha JR, Kuzel P, Garcia E, Persad S (2012) Cadherin switch from E- to N-cadherin in melanoma progression is regulated by the PI3K/PTEN pathway through Twist and Snail. Br J Dermatol 166: 1184–1197. 22332917 10.1111/j.1365-2133.2012.10824.x

[35] Borchers A, David R, Wedlich D (2001) Xenopus cadherin-11 restrains cranial neural crest migration and influences neural crest specification. Development 128: 3049–3060. 11688555 10.1242/dev.128.16.3049

[36] Coen K, Flannagan RS, Baron S, Carraro-Lacroix LR, Wang D, et al (2012) Lysosomal calcium homeostasis defects, not proton pump defects, cause endo-lysosomal dysfunction in PSEN-deficient cells. J Cell Biol 198: 23–35. 22753898 10.1083/jcb.201201076 PMC3392942

[37] Chintapalli VR, Wang J, Dow JA (2007) Using FlyAtlas to identify better Drosophila melanogaster models of human disease. Nat Genet 39: 715–720. 17534367 10.1038/ng2049

[38] Toyomura T, Oka T, Yamaguchi C, Wada Y, Futai M (2000) Three subunit a isoforms of mouse vacuolar H(+)-ATPase. Preferential expression of the a3 isoform during osteoclast differentiation. J Biol Chem 275: 8760–8765. 10722719 10.1074/jbc.275.12.8760

[39] Saw NM, Kang SY, Parsaud L, Han GA, Jiang T, et al (2011) Vacuolar H(+)-ATPase subunits Voa1 and Voa2 cooperatively regulate secretory vesicle acidification, transmitter uptake, and storage. Mol Biol Cell 22: 3394–3409. 21795392 10.1091/mbc.E11-02-0155 PMC3172264

[40] Kawabe H, Sakisaka T, Yasumi M, Shingai T, Izumi G, et al (2003) A novel rabconnectin-3-binding protein that directly binds a GDP/GTP exchange protein for Rab3A small G protein implicated in Ca(2+)-dependent exocytosis of neurotransmitter. Genes Cells 8: 537–546. 12786944 10.1046/j.1365-2443.2003.00655.x

[41] Murphy JE, Padilla BE, Hasdemir B, Cottrell GS, Bunnett NW (2009) Endosomes: a legitimate platform for the signaling train. Proc Natl Acad Sci U S A 106: 17615–17622. 19822761 10.1073/pnas.0906541106 PMC2764915

[42] Dobrowolski R, Vick P, Ploper D, Gumper I, Snitkin H, et al (2012) Presenilin deficiency or lysosomal inhibition enhances Wnt signaling through relocalization of GSK3 to the late-endosomal compartment. Cell Rep 2: 1316–1328. 23122960 10.1016/j.celrep.2012.09.026 PMC3538832

[43] De Calisto J, Araya C, Marchant L, Riaz CF, Mayor R (2005) Essential role of non-canonical Wnt signalling in neural crest migration. Development 132: 2587–2597. 15857909 10.1242/dev.01857

[44] Carmona-Fontaine C, Matthews HK, Kuriyama S, Moreno M, Dunn GA, et al (2008) Contact inhibition of locomotion in vivo controls neural crest directional migration. Nature 456: 957–961. 19078960 10.1038/nature07441 PMC2635562

[45] Wang X, Kopinke D, Lin J, McPherson AD, Duncan RN, et al (2012) Wnt signaling regulates postembryonic hypothalamic progenitor differentiation. Dev Cell 23: 624–636. 22975330 10.1016/j.devcel.2012.07.012 PMC3445042

[46] Medina A, Reintsch W, Steinbeisser H (2000) Xenopus frizzled 7 can act in canonical and non-canonical Wnt signaling pathways: implications on early patterning and morphogenesis. Mech Dev 92: 227–237. 10727861 10.1016/s0925-4773(00)00240-9

[47] Steventon B, Araya C, Linker C, Kuriyama S, Mayor R (2009) Differential requirements of BMP and Wnt signalling during gastrulation and neurulation define two steps in neural crest induction. Development 136: 771–779. 19176585 10.1242/dev.029017 PMC2685944

[48] Villanueva S, Glavic A, Ruiz P, Mayor R (2002) Posteriorization by FGF, Wnt, and retinoic acid is required for neural crest induction. Dev Biol 241: 289–301. 11784112 10.1006/dbio.2001.0485

[49] Gao AC, Lou W, Isaacs JT (1998) Down-regulation of homeobox gene GBX2 expression inhibits human prostate cancer clonogenic ability and tumorigenicity. Cancer Res 58: 1391–1394. 9537237

[50] Smardon AM, Tarsio M, Kane PM (2002) The RAVE complex is essential for stable assembly of the yeast V-ATPase. J Biol Chem 277: 13831–13839. 11844802 10.1074/jbc.M200682200

[51] Kimmel CB, Ballard WW, Kimmel SR, Ullmann B, Schilling TF (1995) Stages of embryonic development of the zebrafish. Dev Dyn 203: 253–310. 8589427 10.1002/aja.1002030302

[52] Thisse C, Thisse B, Schilling TF, Postlethwait JH (1993) Structure of the zebrafish snail1 gene and its expression in wild-type, spadetail and no tail mutant embryos. Development 119: 1203–1215. 8306883 10.1242/dev.119.4.1203

[53] Knight RD, Nair S, Nelson SS, Afshar A, Javidan Y, et al (2003) lockjaw encodes a zebrafish tfap2a required for early neural crest development. Development 130: 5755–5768. 14534133 10.1242/dev.00575

[54] Thisse C, Thisse B (2008) High-resolution in situ hybridization to whole-mount zebrafish embryos. Nat Protoc 3: 59–69. 18193022 10.1038/nprot.2007.514

[55] Matthews HK, Marchant L, Carmona-Fontaine C, Kuriyama S, Larrain J, et al (2008) Directional migration of neural crest cells in vivo is regulated by Syndecan-4/Rac1 and non-canonical Wnt signaling/RhoA. Development 135: 1771–1780. 18403410 10.1242/dev.017350

