# Supplementary figures and images for: Rabconnectin-3a Regulates Vesicle Endocytosis and Canonical Wnt Signaling in Zebrafish Neural Crest Migration
Source: PLoS Biol. 2014 May 6;12(5):e1001852. doi: 10.1371/journal.pbio.1001852 (PMC4011682; doi:10.1371/journal.pbio.1001852)

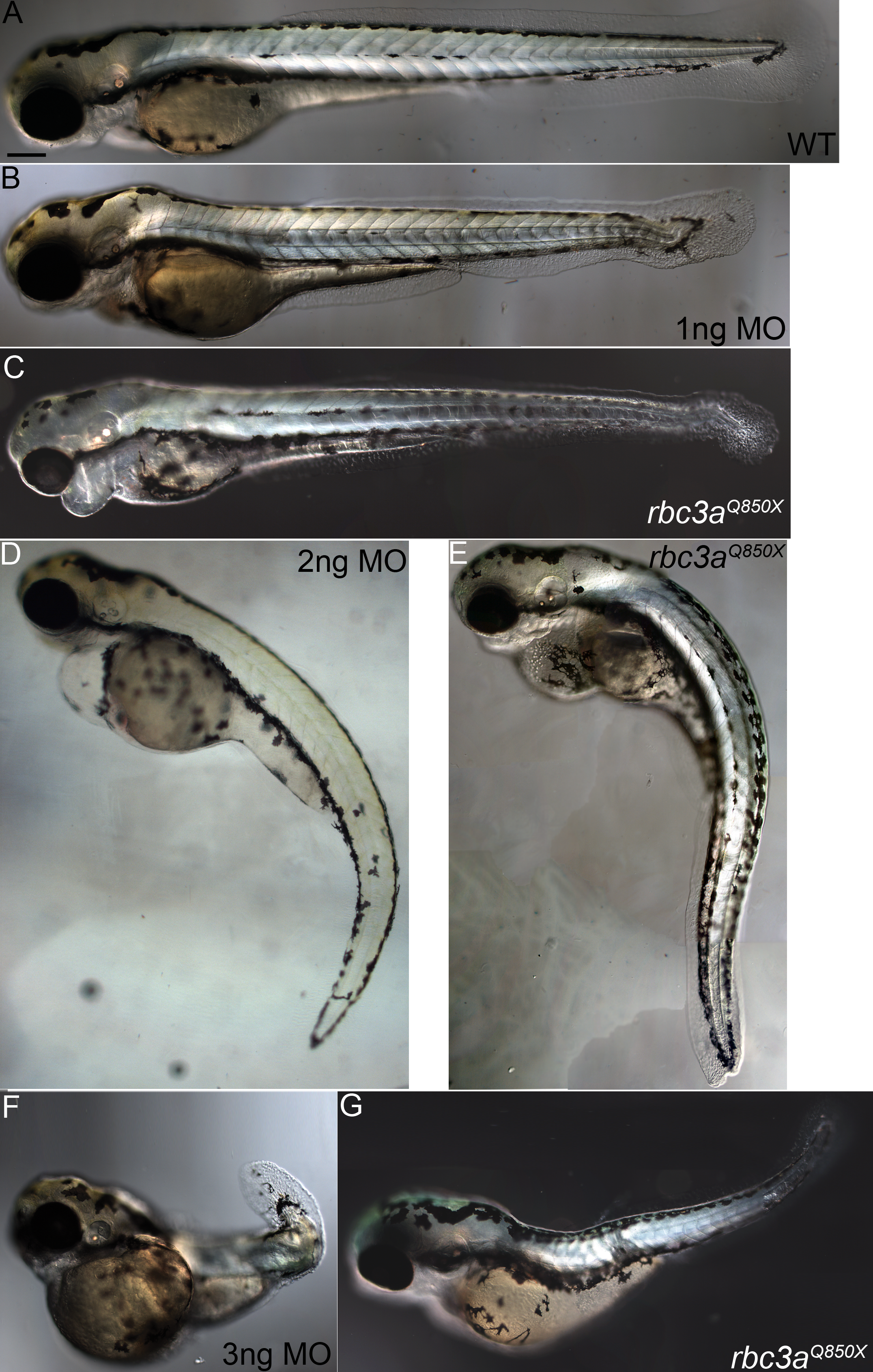

Supplement: Figure S1 — Rbc3a loss-of-function phenotype at 72 hpf. (A, B, D, F) Increasing amounts of rbc3a-MO1 (1–3 ng/embryo) leads to cardiac edema, reduced melanocytes, and shortened, curved tails in larvae at 72 hpf. (C, E, G) rbc3a mutant larvae show similar phenotypes. Scale bar, 200 µm. (TIF) [file pbio.1001852.s001.tif]

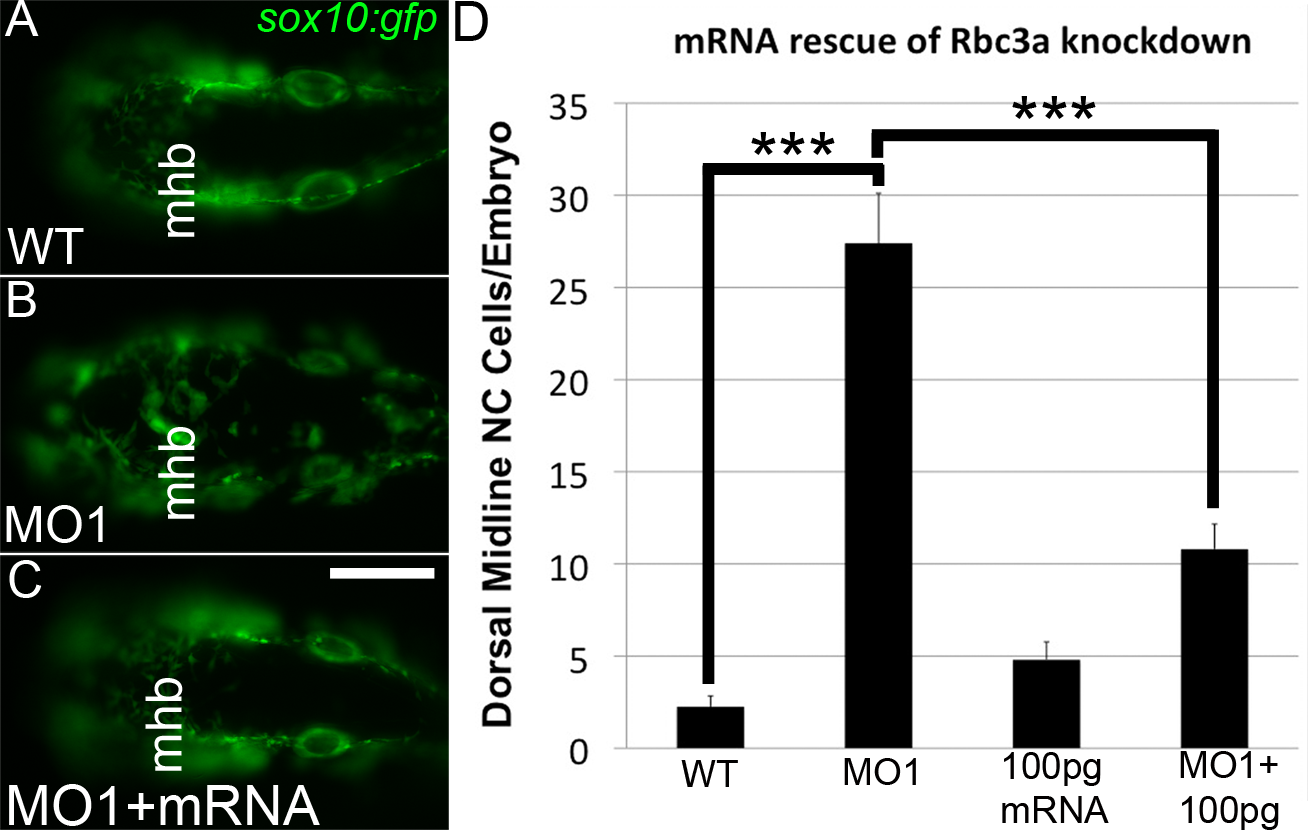

Supplement: Figure S2 — Rescue of rbc3a-MO1–injected embryos with full-length rbc3a mRNA. (A–C) Fluorescent images of live sox10:gfp transgenics, dorsal views, anterior to the left: (A) wild-type (WT), (B) rbc3a-MO1 injected, and (C) co-injected with full length rbc3a mRNA. (D) Number of GFP+ cells aggregated at the dorsal midilne at 24 hpf and located between the otic vesicle and MHB along the A–P axis. Injection of 100 pg rbc3a mRNA significantly rescued the number of GFP+ cells from 27.4±2.7 in rbc3a-MO1–injected embryos (n = 8) to 10.8±3.0 cells in mRNA+MO injected embryos (p<0.001, n = 8). Injection of 100 pg mRNA alone (n = 8) had no effect on the number of midline GFP+ cells compared to 2.3±1.7 in wild-type embryos (n = 9). Error bars represent ± SEM. Scale bar, 100 µm. (TIF) [file pbio.1001852.s002.tif]

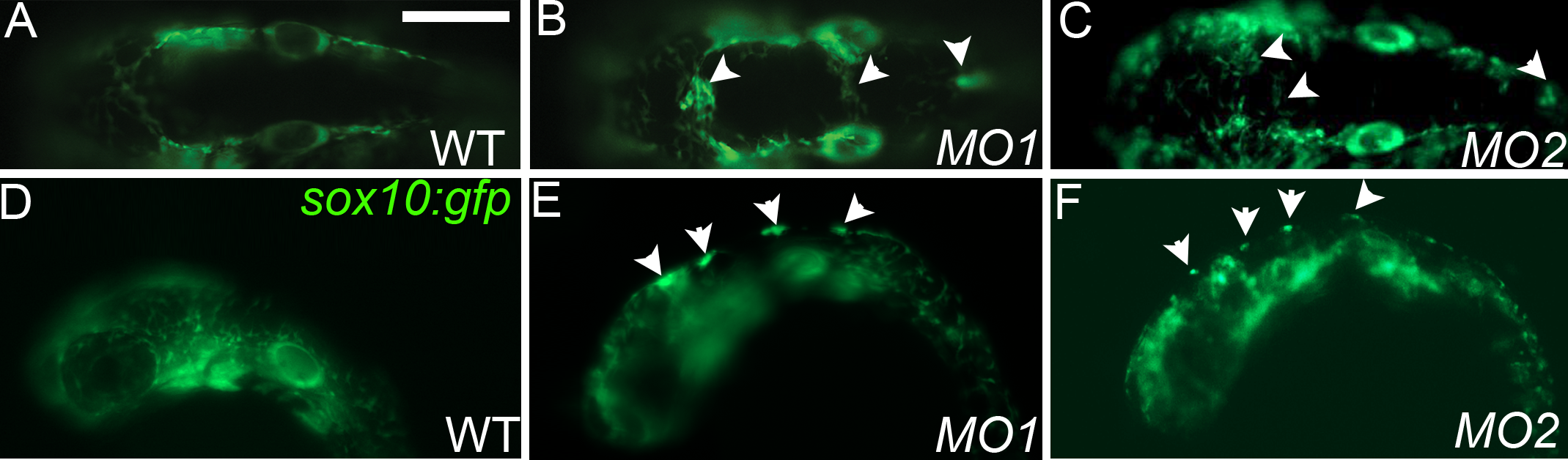

Supplement: Figure S3 — Injection of rbc3a-MO1 or -MO2 produces similar NC defects. (A–F) Live sox10:gfp embryos at 24 hpf, dorsal (A–C) and lateral (D–F) views, anterior to the left. Compared to wild-type embryos (A, D), embryos injected with either rbc3a-MO1 (B, E) or rbc3a-MO2 (C, F) display similar sox10:gfp+ dorsal midline cell aggregates (white arrowheads). (TIF) [file pbio.1001852.s003.tif]

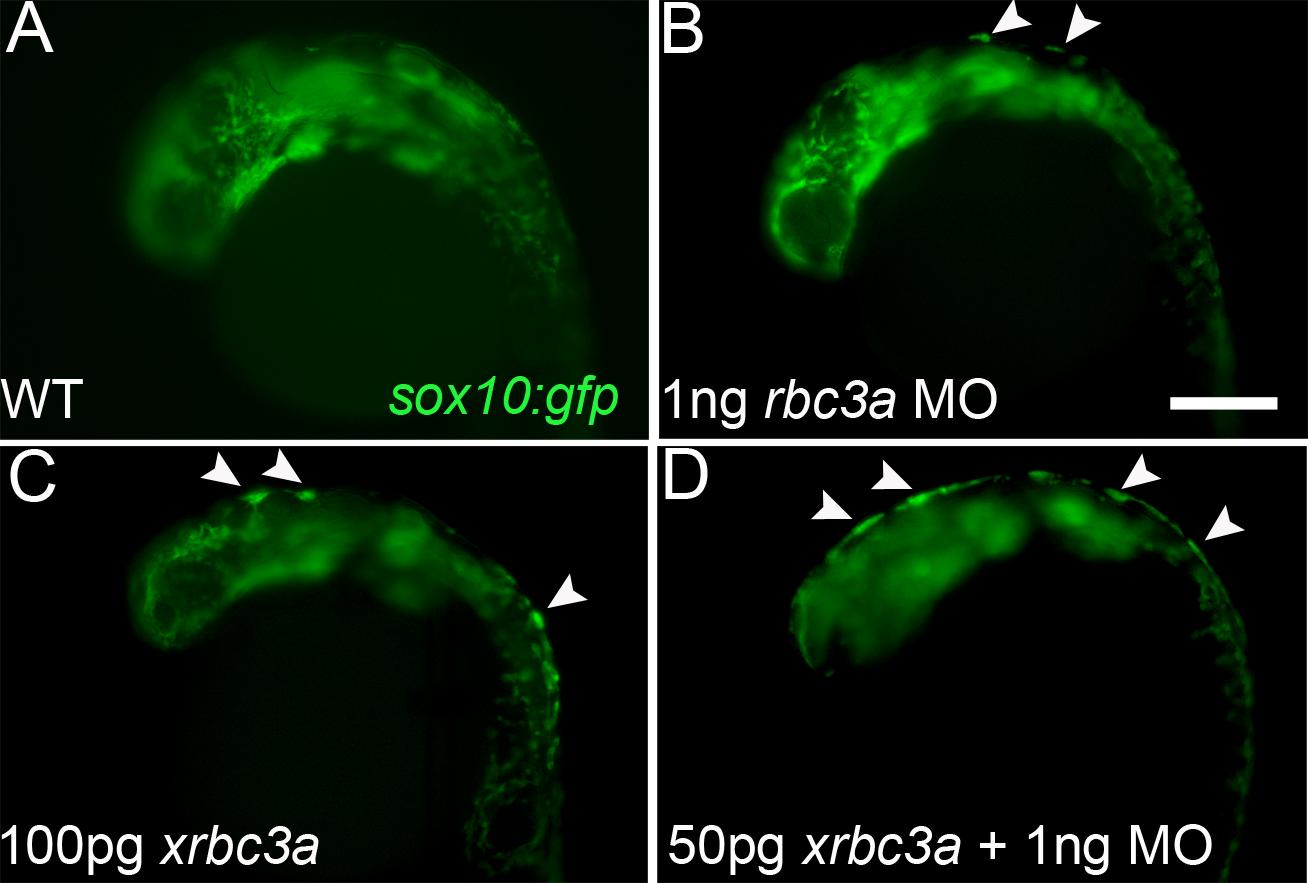

Supplement: Figure S4 — Injection of a 3′-truncated Xenopus rbc3a construct phenocopies Rbc3a loss of function. Fluorescent images of live sox10:gfp transgenics, lateral views, anterior to the left. (A, B) Injection of 1 ng/embryo of rbc3a-MO1 caused GFP+ cells to aggregate at the dorsal midline (arrowheads) by 24 hpf (41%, n = 7/17). (C) Injection of 100 pg/embryo of Xenopus rbc3a mRNA lacking 2.2 kb of the 3′ end of the ORF caused similar GFP+ dorsal aggregates (83%, n = 15/18). (D) Co-injection of 50 pg/embryo of truncated xrbc3a mRNA with 1 ng/embryo of rbc3a-MO1 increased the number and severity of embryos with GFP+ dorsal aggregates (79%, n = 11/14), with some embryos exhibiting a continuous strip of GFP+ cells all along the dorsal midline (36%, n = 5/14). (TIF) [file pbio.1001852.s004.tif]

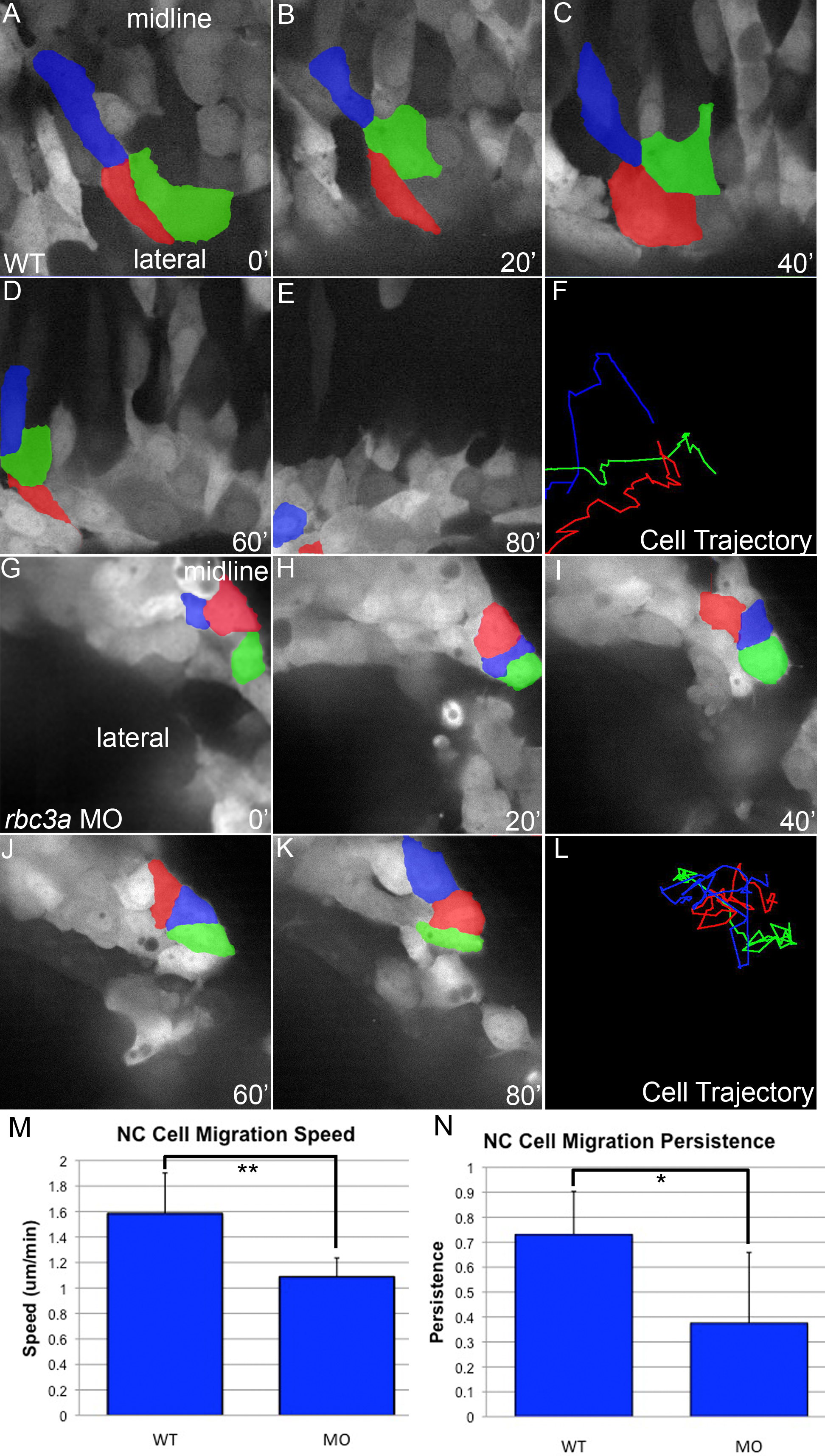

Supplement: Figure S5 — Reduced NC cell motility in rbc3a-MO1–injected embryos. Individual frames from confocal time-lapsed movies of wild-type (A–E) and rbc3a-deficient (G–K) embryos from 13 hpf onwards in 20-min intervals. (F, L) Cell trajectories over 2 h of the corresponding cells in (A, F, and G–K). Wild-type NC cells (F) display stereotypical rapid, directed movement laterally and anteriorly, while many NC cells in rbc3a-MO1–injected embryos (L) adhere to each other and fail to migrate with other NC cells. (M) Average NC cell migration speed and (N) persistence of directionality (measured as the total displacement from the starting position of a cell over the total path length) at the onset of migration in wild-type and rbc3a-MO1–injected embryos (MO). Compared to wild-types, rbc3a-MO1 injection led to significantly reduced migration speed (p = 0.0028, 1.58±0.32 and 1.09±0.15 µm/min, respectively) and persistence (p = 0.015, 0.73±0.17 and 0.38±0.28, respectively). Error bars represent ±SEM. * p<0.05, ** p<0.01. (TIF) [file pbio.1001852.s005.tif]

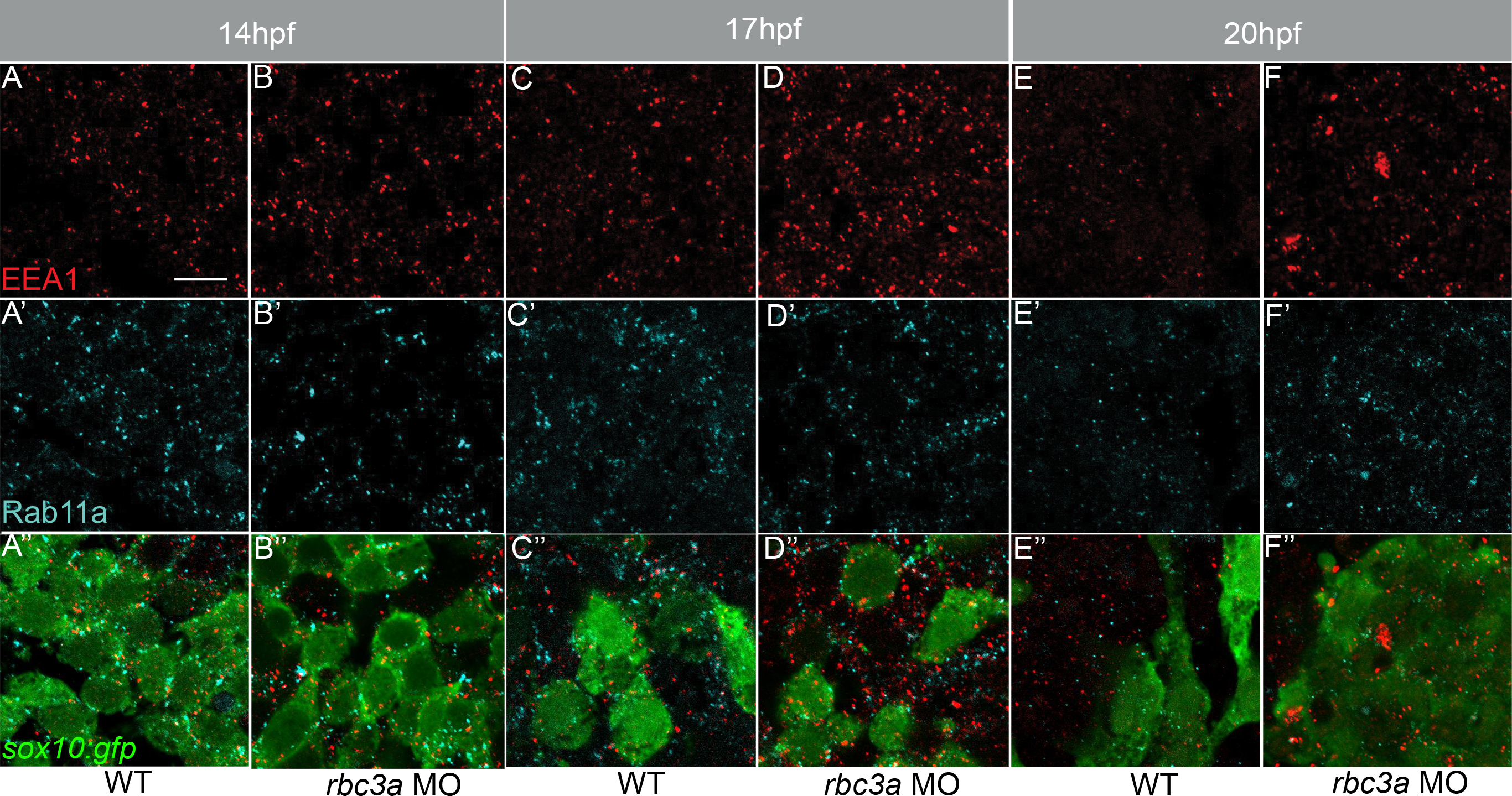

Supplement: Figure S6 — Enlarged early endosomes grow and accumulate over time in NC cells of rbc3a-MO1–injected embryos. Confocal images of sox10:gfp+ NC cells (bottom row, green) double labeled with anti-EEA1 (A–F, red) and anti-Rab11a (A′–F′, blue), which marks late endosomes. (A″–F″) Merged images of EEA1, Rab11a, and sox10:gfp fluorescence. EEA1+ vesicles increase in number and size in rbc3a-MO1–injected embryos from 14–20 hpf compared to wild-type controls, while Rab11a+ vesicles show no change. Scale bars, 10 µm. (TIF) [file pbio.1001852.s006.tif]

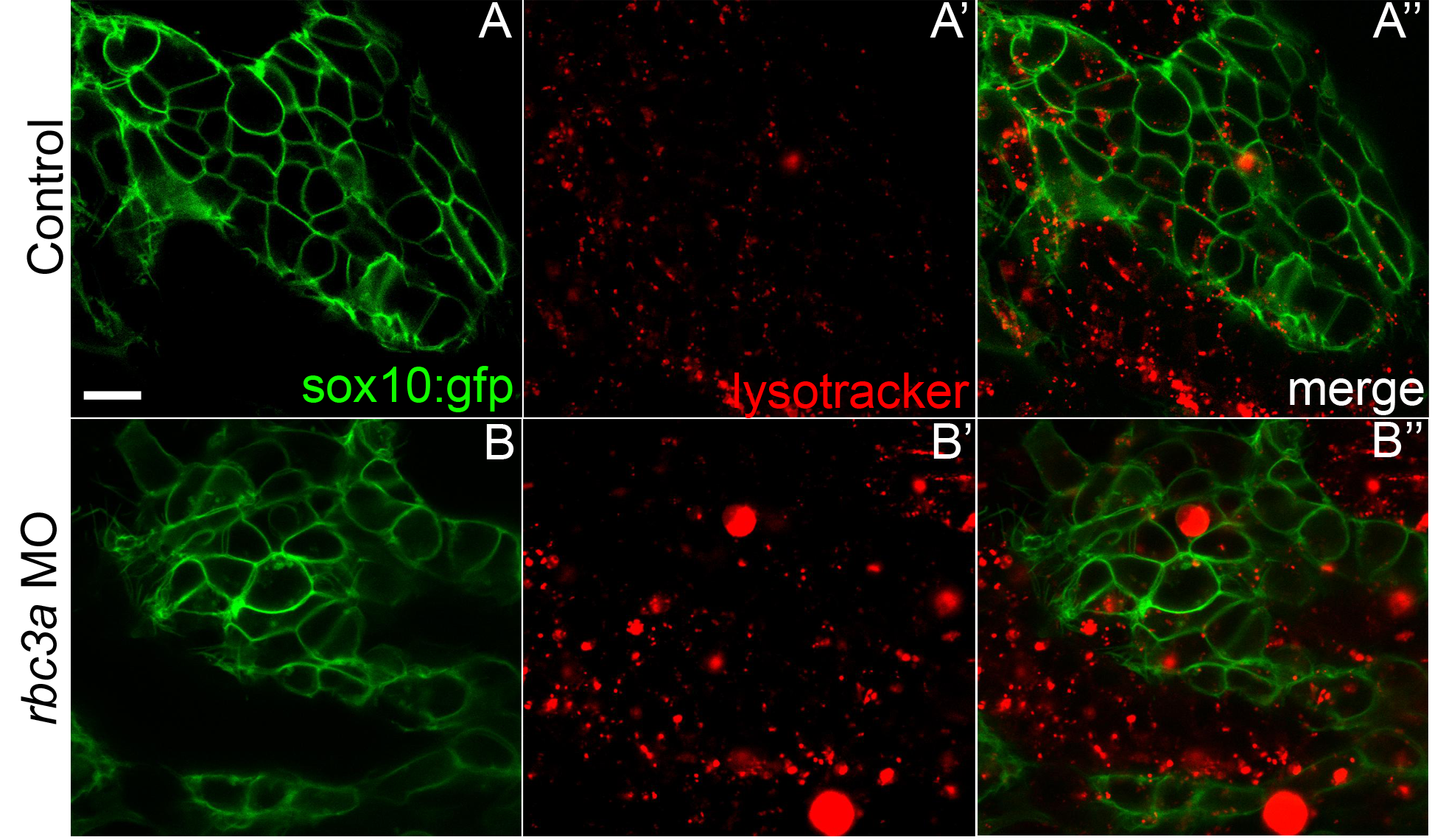

Supplement: Figure S7 — Enlarged early endosomes acidify in NC cells migrating into the pharyngeal arches of rbc3a-MO1–injected embryos. (A–B″) Lateral view of live sox10:lyn-gfp transgenic embryos showing pharyngeal arches stained with Lysotracker-Red. NC cells in rbc3a-MO1–injected embryos contain larger and more Lysotracker-positive intracellular vesicles (B′, B″) compared to controls. Scale bar, 10 µm. (TIF) [file pbio.1001852.s007.tif]

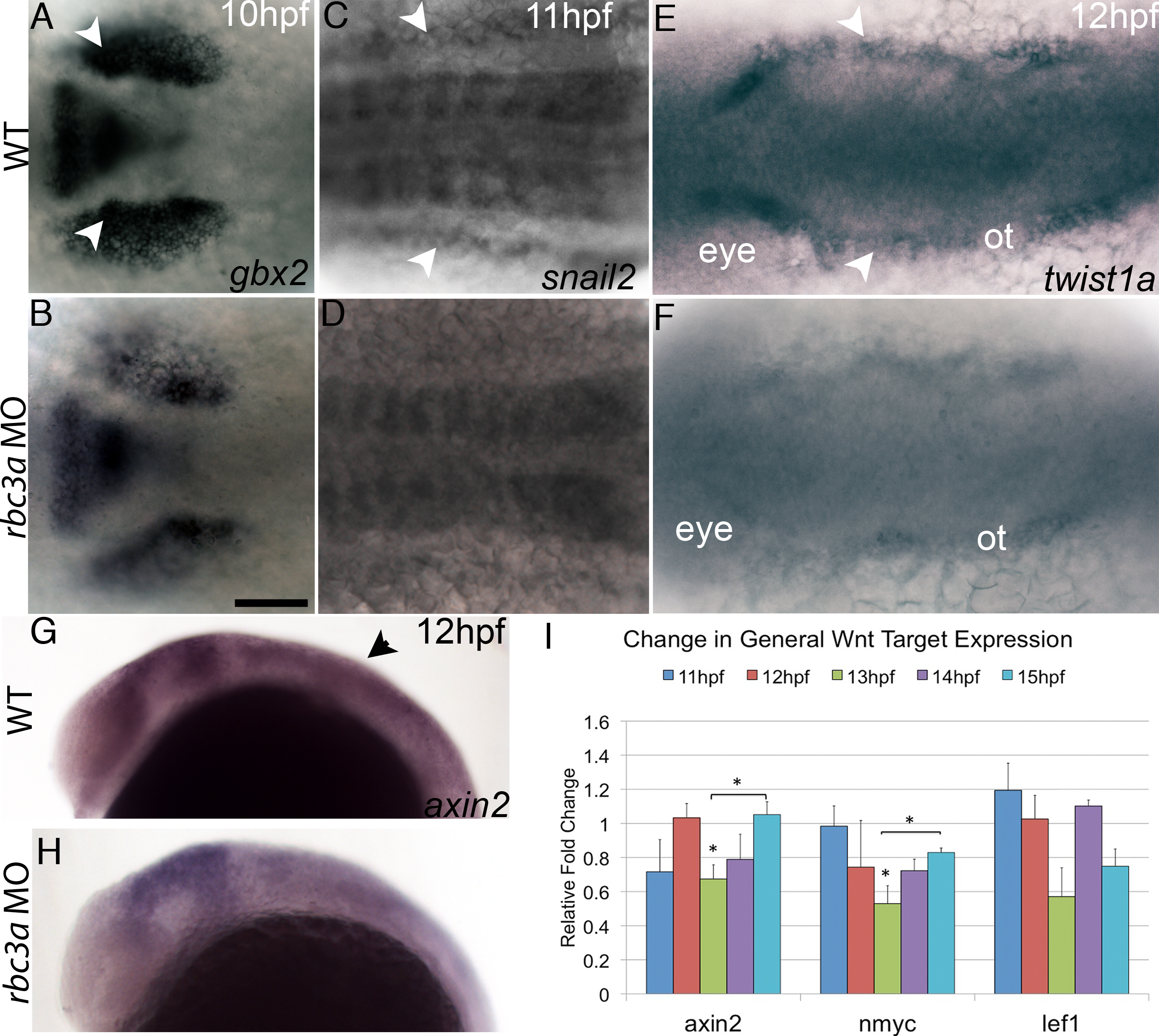

Supplement: Figure S8 — Changes in downstream Wnt target gene expression. (A–H) In situ hybridization for Wnt target gene expression in wild-type and rbc3a-MO1–injected embryos. Dorsal views (A–F), lateral views (G, H). Several genes display reduced expression in the NC (arrowheads) in rbc3a-MO1–injected embryos from 10–12 hpf including gbx2 (A, B), snail2 (C, D), twist1a (E, F), and axin2 (G, H). Scale bar, 100 µm. (I) axin2, nmyc, and lef1 show decreased expression by 13 hpf in rbc3a-MO1–injected embryos. Error bars represent triplicate experiments ± SEM. * p<0.05. (TIF) [file pbio.1001852.s008.tif]

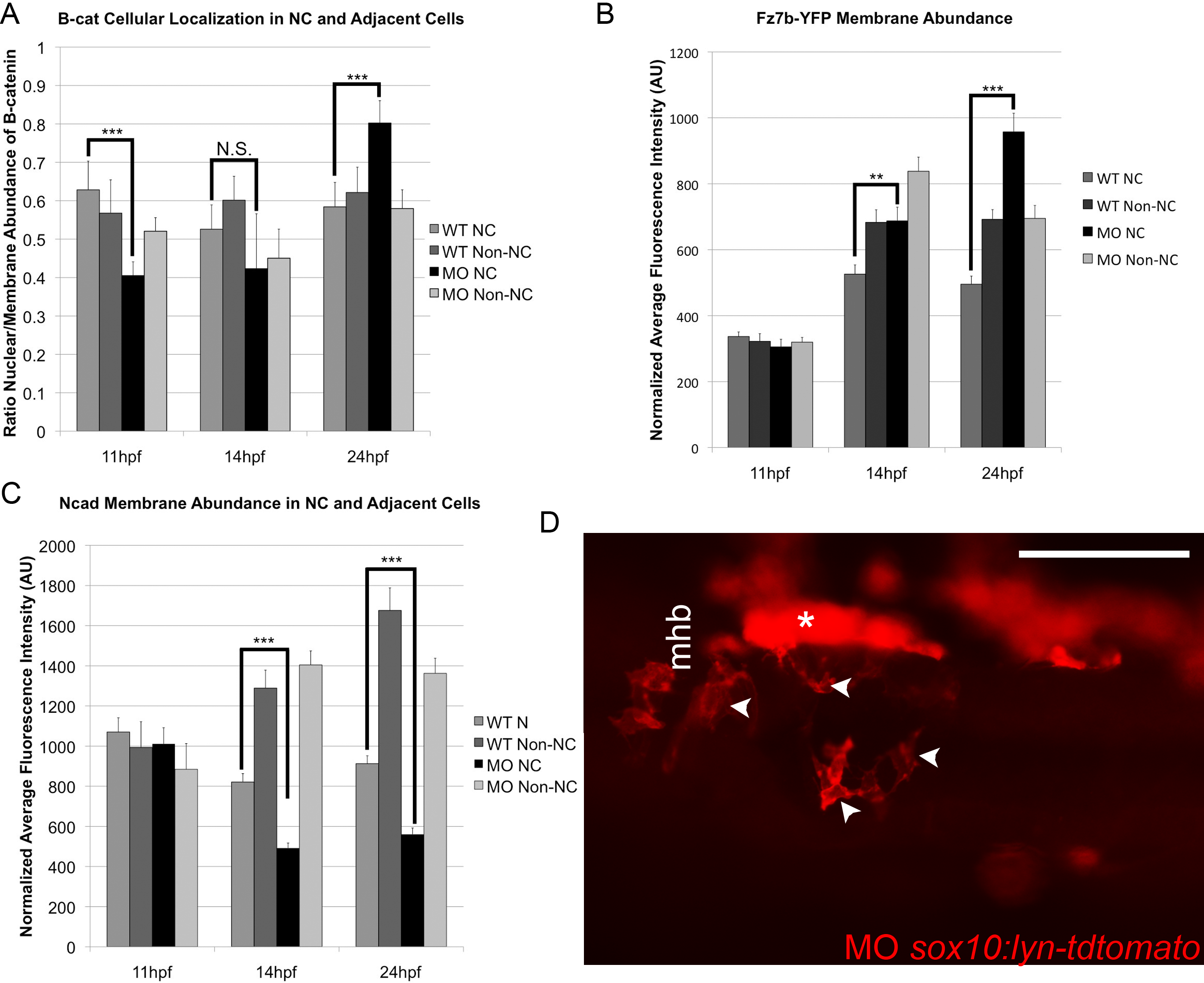

Supplement: Figure S9 — Cell autonomous effects of Rbc3a knockdown. (A–C) Quantification of subcellular localization of (A) Bcat, (B) Fz7b-YFP, and (C) Ncad in NC and non-NC cells. ** p<0.01, *** p<0.001, N.S., not significant. (D) Wild-type host embryo at 24 hpf with sox10:lyn-tdtomato cells (red) transplanted from a rbc3a-MO1–injected donor, dorsal view. A subset of rbc3a-deficient donor cells formed dorsal midline aggregates (white arrowheads), but many other cells migrated properly into the pharyngeal arches (asterisk). Mhb, mindbrain-hindbrain boundary. Scale bar, 100 µm. (TIF) [file pbio.1001852.s009.tif]

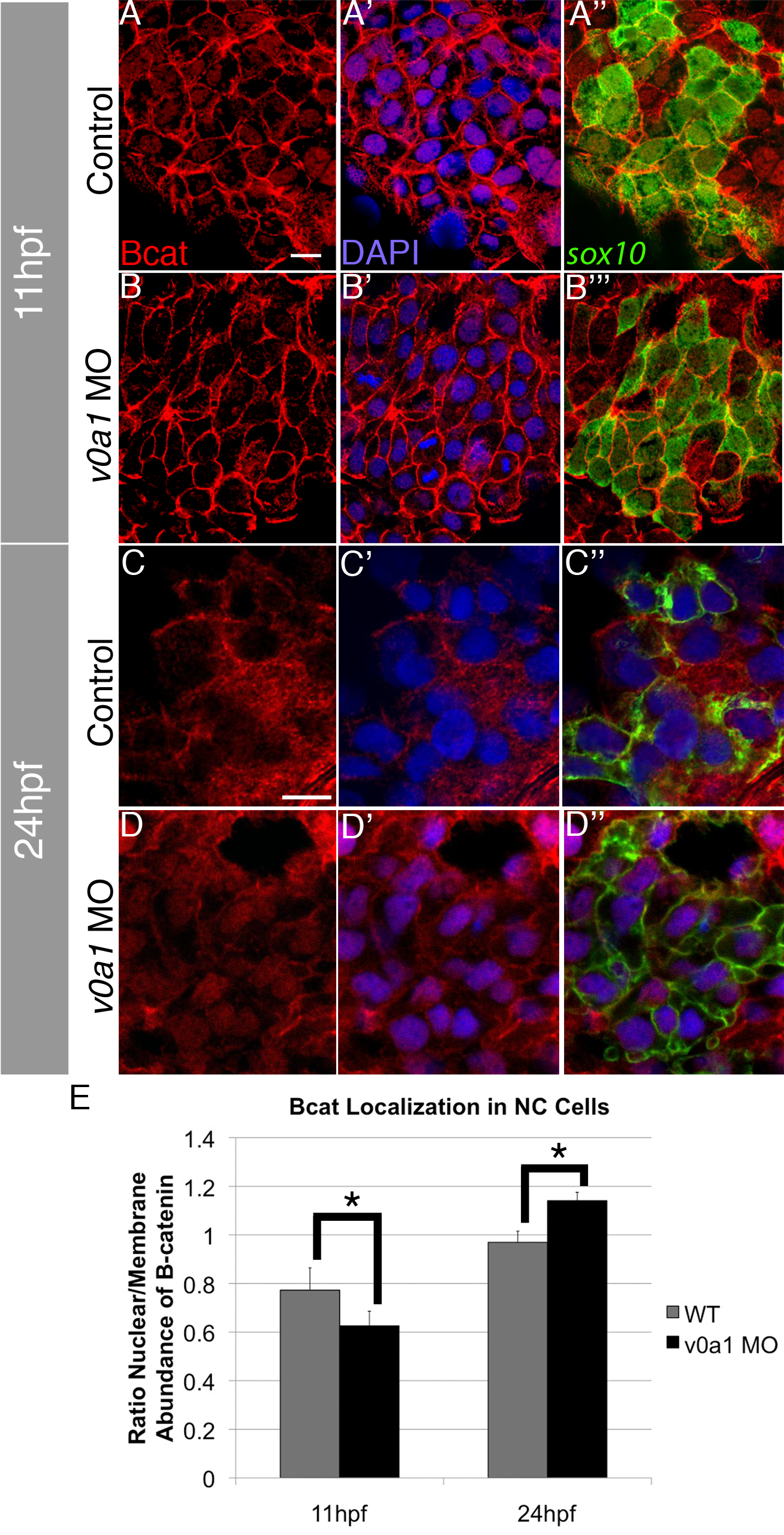

Supplement: Figure S10 — V0a1 knockdown disrupts nuclear localization of Bcat in NC. (A–D″) Confocal images of sox10:gfp (green) embryos stained with DAPI (blue) and an anti-Bcat antibody (red) in uninjected controls (A–A″, C–C″) and embryos injected with v0a1-MO at 11 hpf (B–B″) and 24 hpf (D–D″). V0a1 knockdown reduces levels of B-cat in NC cell nuclei at 11 hpf (B–B″) and nuclear Bcat at 24 hpf (D–D″). Scale bars, 10 µm. (E) Quantification of the ratio of Bcat localization between NC cell membranes and nuclei indicates a significant decrease in nuclear localization in rbc3a-MO1–injected embryos at 11 hpf but a significant increase in nuclear localization at 24 hpf. Errors bars respresent ± SEM, * p<0.05. (TIF) [file pbio.1001852.s010.tif]
