# Supplementary material for: Rabconnectin-3a Regulates Vesicle Endocytosis and Canonical Wnt Signaling in Zebrafish Neural Crest Migration
Source: PLoS Biol. 2014 May 6;12(5):e1001852. doi: 10.1371/journal.pbio.1001852 (PMC4011682; doi:10.1371/journal.pbio.1001852)
Supplement: Table S1 — Similar dorsal midline aggregates of NC cells following rbc3a-MO2 injection further confirm morpholino specificity. Both control and rbc3a mRNA-injected embryos show no NC defects. In contrast, rbc3a-MO2–injected embryos display dorsal midline NC cells (85.3%) compared to control embryos (p≪0.0001). Co-injection of rbc3a mRNA with rbc3a-MO2 partially rescues these NC defects (47.3%; p≪0.0001). (DOCX) [file pbio.1001852.s013.docx]

**Table S1: Similar dorsal midline aggregates of neural crest cells following *rbc3a*-MO2 injection further confirm morpholino specificity.**

| **Treatment** | **NC phenotype** | **Wild-type** | **Total** |
| --- | --- | --- | --- |
| Control | 0 | 27 | 27 |
| 200 pg mRNA | 1 | 17 | 18 |
| *rbc3a*-MO2 | 35 | 6 | 41 |
| MO2+mRNA rescue | 10 | 11 | 21 |
