# Supplementary material for: Rabconnectin-3a Regulates Vesicle Endocytosis and Canonical Wnt Signaling in Zebrafish Neural Crest Migration
Source: PLoS Biol. 2014 May 6;12(5):e1001852. doi: 10.1371/journal.pbio.1001852 (PMC4011682; doi:10.1371/journal.pbio.1001852)
Supplement: Table S2 — Antisense MO design. (DOCX) [file pbio.1001852.s014.docx]

**Table S2. Antisense Morpholino Oligonucleotide (MO) Design**

| **MO Name** | **Sequence (5’-3’)** |
| --- | --- |
| *rbc3a*-MO | CTTGTTTCCCTTCTCCAATTTCCAT |
| *rbc3a*-MO2 | CATTTTTGTTGATCACGCCGATGTT |
| *atp6v0a1-*MO (*v0a1­*-MO) | GAAATGGTCTGCACTTACATCTCTG |
| *p53*-MO | AGAATTGATTTTGCCGACCTCCTCT |
