# Supplementary material for: Rabconnectin-3a Regulates Vesicle Endocytosis and Canonical Wnt Signaling in Zebrafish Neural Crest Migration
Source: PLoS Biol. 2014 May 6;12(5):e1001852. doi: 10.1371/journal.pbio.1001852 (PMC4011682; doi:10.1371/journal.pbio.1001852)
Supplement: Table S3 — Primer sequences: 5′-3′ sequences of qPCR and probe synthesis primers. (DOCX) [file pbio.1001852.s015.docx]

**Table S3. Primer Sequences**.

| **Primer** | **Sequence (5’ to 3’)** |
| --- | --- |
| *axin2 F* | GGACACTTCAAGGAACAACTAC |
| *axin2 R* | CCTCATACATTGGCAGAACTG |
| *lef1 F* | GAGGGAAAAGATCCAGGAAC |
| *lef1 R* | AGGTTGAGAAGTCTAGCAGG |
| *gbx2 F* | AAACGTTTGCGACGAAAGAT |
| *gbx2 R* | CAAAGACGACTCGAAGGAG |
| *snai2 F* | TGAACTGGAGAGTCCGACAG |
| *snai2 R* | GCAGGTTGCTGGTAGTCCAT |
| *mycn F* | AACAAGAGGGAGAATGCCA |
| *mycn R* | TAGAAGTCATCCTCGTCCG |
| *ecad F* | GGGATTTCTGTGCTGGAAAA |
| *ecad R* | TTGACAAACCCTCTCCGTTC |
| *ncad F* | TGTGAATCGCGTGAAAAGAG |
| *ncad R* | AGCGTGTTGCTCTTGTCCTT |
| *cdh11 F* | GGAAGACATTCGGGAGAACA |
| *cdh11 R* | TGTAAGGTGGCGATGTCAAA |
| *ef1a F* | CAAGGGATGGAAGATTGAGC |
| *ef1a R* | AACCATACCAGGCTTGAGGA |
| *twist1a* F | TACCCTCGGACAAACTCAGC |
| *twist1a* R | GGACCTGACAGAGGAAGTCAA |
| *mitfa* F | CAACTGTGAGAAAGAGATGGA |
| *mitfa R* | AACCCAAGAATGTCATCACTG |
| **Additional Primers** | |
| *rbc3a probe F* | ACAGCGCTGATGAAATAAAC |
| *rbc3a probe R* | TAATACGACTCACTATAGGGAGGAGTTGGAGGAGGAAC |
