# Supplementary material for: Rabconnectin-3a Regulates Vesicle Endocytosis and Canonical Wnt Signaling in Zebrafish Neural Crest Migration
Source: PLoS Biol. 2014 May 6;12(5):e1001852. doi: 10.1371/journal.pbio.1001852 (PMC4011682; doi:10.1371/journal.pbio.1001852)
Supplement: Table S4 — Antibody staining sources and conditions. (DOCX) [file pbio.1001852.s016.docx]

**Table S4. Antibody Staining Sources and Conditions.**

| **Primary Antibody** | **Raised In** | **Source** | **Concentration** |
| --- | --- | --- | --- |
| α-GFP | Chicken | Abcam | 1:1000 |
| α-EEA1 | Rabbit | Abcam | 1:200 |
| α-LAMP1 | Rabbit | Abcam | 1:200 |
| α-Rab11a | Mouse | Abcam | 1:200 |
| α-N-cadherin | Rabbit | Genetex | 1:200 |
| α-B-catenin | Mouse | Genetex | 1:200 |
| **Secondary Antibody** | **Raised in** | **Source** | **Concentration** |
| α-Rabbit-Cy3 | Donkey | Jackson | 1:1000 |
| α-Rabbit-Cy5 | Donkey | Jackson | 1:1000 |
| α-Mouse-AlexaFluor568 | Goat | Life | 1:1000 |
| α-Mouse-Cy5 | Goat | Life | 1:1000 |
| α-chicken-AlexaFluor488 | Donkey | Jackson | 1:1000 |
